# Compartment-dependent mitochondrial alterations in experimental ALS, the effects of mitophagy and mitochondriogenesis

**DOI:** 10.3389/fncel.2015.00434

**Published:** 2015-11-06

**Authors:** Gianfranco Natale, Paola Lenzi, Gloria Lazzeri, Alessandra Falleni, Francesca Biagioni, Larisa Ryskalin, Francesco Fornai

**Affiliations:** ^1^Department of Translational Research and New Technologies in Medicine and Surgery, University of PisaItaly; ^2^Department of Clinical and Experimental Medicine, University of PisaItaly; ^3^I.R.C.C.S., NeuromedPozzilli, Italy

**Keywords:** mitochondria, amyotrophic lateral sclerosis, autophagy, lithium, G93A transgenic mouse, motor neuron, electron microscopy, biogenesis of mitochondria

## Abstract

Amyotrophic lateral sclerosis (ALS) is characterized by massive loss of motor neurons. Data from ALS patients and experimental models indicate that mitochondria are severely damaged within dying or spared motor neurons. Nonetheless, recent data indicate that mitochondrial preservation, although preventing motor neuron loss, fails to prolong lifespan. On the other hand, the damage to motor axons plays a pivotal role in determining both lethality and disease course. Thus, in the present article each motor neuron compartment (cell body, central, and peripheral axons) of G93A SOD-1 mice was studied concerning mitochondrial alterations as well as other intracellular structures. We could confirm the occurrence of ALS-related mitochondrial damage encompassing total swelling, matrix dilution and cristae derangement along with non-pathological variations of mitochondrial size and number. However, these alterations occur to a different extent depending on motor neuron compartment. Lithium, a well-known autophagy inducer, prevents most pathological changes. However, the efficacy of lithium varies depending on which motor neuron compartment is considered. Remarkably, some effects of lithium are also evident in wild type mice. Lithium is effective also *in vitro*, both in cell lines and primary cell cultures from the ventral spinal cord. In these latter cells autophagy inhibition within motor neurons *in vitro* reproduced ALS pathology which was reversed by lithium. Muscle and glial cells were analyzed as well. Cell pathology was mostly severe within peripheral axons and muscles of ALS mice. Remarkably, when analyzing motor axons of ALS mice a subtotal clogging of axoplasm was described for the first time, which was modified under the effects of lithium. The effects induced by lithium depend on several mechanisms such as direct mitochondrial protection, induction of mitophagy and mitochondriogenesis. In this study, mitochondriogenesis induced by lithium was confirmed *in situ* by a novel approach using [2-^3^H]-adenosine.

## Introduction

Amyotrophic lateral sclerosis (ALS) is characterized by progressive neurodegeneration of motor neurons within spinal cord, brainstem, and motor cortex (Charcot, 1874; Boillée et al., 2006). This may occur either sporadically (sALS) or depending on a number of gene mutations with familial transmission (fALS), all these mutations leading to autophagy impairment (Pasquali et al., 2014; Cirulli et al., 2015). Twenty percent of fALS occurs due to mutations in the gene coding for the enzyme copper-zinc superoxide dismutase (SOD-1, Rosen et al., 1994), which produces mitochondrial toxicity (Vehviläinen et al., 2014) while being an autophagy substrate (Kabuta et al., 2006). Both fALS and sALS are characterized by severe mitochondrial alterations. In fact, altered mitochondria within motor neurons of ALS patients were originally demonstrated by using electron microscopy (Hart et al., 1977; Hirano et al., 1984a,b; Sasaki, 2011; Ruffoli et al., 2015). Mitochondrial alterations represent a milestone within degenerating motor neurons in ALS. This may occur either primarily, through direct neurotoxicity of mutated proteins toward specific mitochondrial targets (Martin et al., 2007; Vehviläinen et al., 2014), or it may be produced by a defective clearance of altered mitochondria (impaired autophagy and mitophagy).

The occurrence of defective autophagy in ALS was demonstrated for the first time in the last decade (Fornai et al., 2008a,b); and it was progressively validated through a growing number of studies (Pasquali et al., 2009; Ferrucci et al., 2010; Otomo et al., 2012; Shimada et al., 2012; Wang et al., 2012; Castillo et al., 2013; Ikenaka et al., 2013; Barmada et al., 2014; Cheng et al., 2015; Lee et al., 2015; Philips and Rothstein, 2015; Xiao et al., 2015; Yang et al., 2015) up to the very recent confirmative work by Xie et al. (2015a,b). This evidence was reviewed in this issue by Ruffoli et al. (2015). At the same time, within ALS mitochondria an altered calcium buffering activity is constantly described (Higgins et al., 2002; von Lewinski and Keller, 2005; Jaiswal, 2013). This was intensely investigated in the past two decades to elucidate the process of primary mitochondrial damage (Barrett et al., 2014; Vehviläinen et al., 2014), which still requires a prompt mitochondrial removal (mitophagy; Fornai et al., 2008a; Laird et al., 2008; Pasquali et al., 2009; Ruffoli et al., 2015) as well as the synthesis of novel mitochondria (Fornai et al., 2008a,b; Melser et al., 2015; Roy et al., 2015; Ruffoli et al., 2015). A few years ago we demonstrated that, when removal of aged mitochondria within motor neurons is promoted through the autophagy machinery (mitophagy), a concomitant stimulation of the biogenesis of novel mitochondria takes place (Fornai et al., 2008a,b). This is now explained mechanistically by the recent work of Palikaras et al. (2015a,b) who unraveled a specific signaling pathway which binds mitophagy to mitochondriogenesis. Thus, an increase of mitochondrial removal eventually leads to the biogenesis of novel mitochondria (Palikaras et al., 2015a,b). This provides the molecular mechanism explaining why lithium, which is a powerful autophagy (and mitophagy) inducer (Sarkar et al., 2005; Sarkar and Rubinsztein, 2006; Pasquali et al., 2010; Klionsky et al., 2012; Motoi et al., 2014) concomitantly stimulates mitochondriogenesis (Fornai et al., 2008a,b), while protecting against primary mitochondrial damage (Bachmann et al., 2009) and excitotoxicity in the spinal cord (Young, 2009; Calderó et al., 2010; Fulceri et al., 2011). Despite mitochondria are key in the course of motor neuron degeneration in ALS, recent data demonstrate that, when mitochondria are protected in ALS mice, the time course of motor palsy and lethality is not modified (Parone et al., 2013). These data were obtained by suppressing the synthesis of cyclophilin D (CycD-KO), which contributes to the opening of the mitochondrial permeability transition pore. These CycD-KO mice have been generated in different strains of SOD-1 transgenic ALS mice. In all these ALS mice knocking out CycD preserves mitochondria and prevents the loss of motor neurons in the spinal cord. However, no improvement of motor symptoms and survival is obtained (Parone et al., 2013). This is due to the ongoing loss of peripheral motor innervation which persists despite the preservation of motor neuron perikaria.

Unexpectedly, in CycD-KO mice axonal mitochondria were protected as it occurred upstream in the cell body despite muscle denervation persisted. However, mitochondrial preservation within motor axons of CycD-KO mice was measured by Parone et al. (2013) only within ventral roots (proximal axons), while motor axon degeneration was measured at peripheral level within denervated muscles (distal axons). This leaves open the chance that mitochondrial damage within peripheral axons still occurs compared with proximal axons and cell bodies. This calls for additional studies to document the ultrastructure of distal (peripheral) compared with proximal axons before ruling out the protective role of mitochondrial preservation. In fact, it may occur that mitochondrial integrity measured in the ventral root is no longer present in peripheral axons. On the other hand, if mitochondrial alterations in distal axons are prevented despite an ongoing muscle denervation, alternative hypothesis of pathological targets need to be taken into account. In this context, altered axonal structure beyond mitochondria needs to be investigated. In recent years, the axonal transport was shown to be impaired in ALS. This may lead to abnormal accumulation of organelles and protein aggregates. In this way, a combined defect in axonal transport including mitochondria may converge in determining axonal damage. For instance, a specific impairment of axonal transport for mitochondria in G93A mice was clearly documented (Magrané et al., 2012, 2014). Since mitochondria are mostly synthesized at the level of the cell body, this may lead to increasing deficit of mitochondria along axonal length. This is expected to produce the most severe mitochondrial reduction at the level of the most remote segments of distal axons. If this is the case, apart from mitochondrial protection, the presence of mitochondria, comparing proximal with distal axons, should be evaluated.

In any case, motor axon degeneration represents a dominant effect compared with the loss of cell bodies in the course of ALS. Similarly, the ultimate anatomical target to promote neuroprotection in ALS shifts from the spinal cord to the muscle. This leads to focus on diverse motor neuron compartments (primarily distal axons compared with proximal axons, cell body, and dendrites).

Therefore, in the present study ultrastructural morphometry of compartment-dependent mitochondrial alterations occurring in G93A mice was carried out. In detail, we used quantitative ultrastructural morphometry to count mitochondrial alterations occurring at the level of dendrites, neuronal cell bodies, and motor axons. This latter compartment was further evaluated to measure mitochondrial alterations within distal compared with proximal axons. We examined at transmission electron microscopy (TEM) changes of mitochondrial size, amount, distribution, and mitochondrial alterations involving selective organelle architecture and swelling. Moreover, to understand the phenomena of axonal degeneration beyond mitochondrial level we investigated the fine ultrastructure of additional sub-cellular components within ALS proximal and distal motor axons. This was measured both in baseline conditions, in G93A SOD-1 transgenic mice compared with wild types, and following lithium to produce autophagy stimulation (Sarkar et al., 2005; Sarkar and Rubinsztein, 2006; Fornai et al., 2008a; Pasquali et al., 2010; Klionsky et al., 2012; Motoi et al., 2014). In fact, lithium is known for a long time as a mood stabilizer and it was recently shown to possess neuroprotective effects in various degenerative disorders, including those affecting motor neurons (Madeo et al., 2009; Luo, 2010; Shimada et al., 2012; Chiu et al., 2013; Agam and Israelson, 2014; Lieu et al., 2014; Mohammadianinejad et al., 2014; Motoi et al., 2014; Yáñez et al., 2014; Dwivedi and Zhang, 2015). This is in line with recent data showing that all autophagy inducers being tested so far protect in ALS experimental models. This is the case of rapamycin (Wang et al., 2012; Cheng et al., 2015), trehalose (Schaeffer et al., 2012; Castillo et al., 2013; He et al., 2015), resveratrol (Han et al., 2012), spermidine (Wang et al., 2012), tamoxifene (Wang et al., 2012), carbamazepine (Wang et al., 2012), valproate (Boll et al., 2014; Wang et al., 2015), histone deacetylase 6 (Chen et al., 2015), food starvation (Wiesner et al., 2015). This was also achieved by increasing expression of autophagy activating genes (Hetz et al., 2009; Oliván et al., 2015; Yang et al., 2015). Lithium administration may not induce autophagy if lithium plasma levels are way below those required to stimulate the autophagy pathway and produce any therapeutic effect (Pizzasegola et al., 2009; Chiu et al., 2013). Since, lithium-induced ultrastructural changes produce marked effects on mitochondria, involving size, shape, number and architecture, we further detailed these effects *in vitro* in cell lines to reduce experimental variables. In these experimental conditions, we could replicate the effects observed *ex vivo* in the spinal cord and we set up the ultrastructural measurement of mitochondriogenesis. In particular, we wish to provide for the first time *in situ* morphological evidence for mitochondriogenesis by high resolution (TEM) autoradiography of [2-^3^H]adenosine.

## Materials and methods

### *Ex Vivo* study

#### Animals

Male B6SJL-TgN (SOD1-G93A)1 Gur mice, expressing human G93A SOD-1 mutation (*N* = 10) and wild type (WT) littermates (*N* = 10) were purchased from the Jackson Laboratory (Bar Harbor, ME, USA) via Charles River (Calco, LC, Italy). Mice received food and water *ad libitum* and were housed under controlled conditions: 12 h light/dark cycle, 21°C room temperature.

This study was carried out in agreement with the European Council directive (86/609/EEC). The experiments were approved by the Ethical Committee at the University of Pisa (protocol n. 14540, November, 21st, 2011).

#### Experimental groups and treatments

Transgenic G93A SOD-1 mice and their WT littermates were divided into four experimental groups: lithium-treated G93A mice (*N* = 5); vehicle (saline, sodium chloride 0.9%)-treated G93A mice (*N* = 5); lithium-treated WT mice (*N* = 5) and vehicle-treated WT mice (*N* = 5). All treatments started at 67 days of age, which corresponds to a pre-symptomatic stage, and they were carried out every other day up to a tetraplegic stage (end point), when mice were sacrificed with deep anesthesia (chloral hydrate). The tetraplegic stage was defined when mice were no longer able to get up from a lying position within a 30 s time interval (Parone et al., 2013; Fornai et al., 2014). This end point was chosen in order to avoid discomfort due to impaired feeding, drinking and breathing (Tankersley et al., 2007; Fornai et al., 2014). Lithium chloride (Sigma, St. Louis, MO, USA) was administered (both to G93A and WT mice) at the dose of 1 mEq/Kg, i.p. dissolved in 200 μl of saline. All treatments were carried out between 9.00 and 12.00 am.

#### Transmission electron microscopy of spinal cord and muscle

Following deep anesthesia mice were trans-cardially rinsed with saline solution (0.9%) and they were fixed by perfusion with 2% paraformaldehyde/0.1% glutaraldehyde in 0.1 M phosphate buffered saline, pH = 7.4. The spinal cord and gastrocnemius muscle were dissected. Spinal cords were kept overnight at 4°C *in situ* using the same fixing solution.

#### Specimens from spinal cord

The lumbar tract of the spinal cord was surgically dissected to avoid any abnormal pressure and post-fixed in a 1% OsO_4_ buffered solution for 1 h and 30 min, and dehydrated in ethanol, and embedded in Epon-araldite.

For each spinal cord sample two tissue blocks (volume of 5 mm^3^) were cut to obtain an average of 20 grids. Each grid included at least 5 cells, which were analyzed along non-serial sections. Motor neurons were selected based on classic morphological features (multipolar cells with dispersed nuclear chromatin and prominent nucleoli). In order to improve the selection of motor neurons we also applied a size exclusion criterion. This consists of excluding from counts those lamina IX neurons measuring less than 30 μm of maximum diameter. This limits the analysis to phasic alpha motor neurons ruling out gamma motor neurons and most tonic alpha motor neurons, but it guarantees to rule out type I Golgi projecting neurons. This size-exclusion criterion is validated by several previous studies adjusted to various mouse strains (Morrison et al., 1998; Martin et al., 2007; Fornai et al., 2008a, 2014; Ferrucci et al., 2010; Fulceri et al., 2012).

Morphometry of motor neurons was carried out by using TEM at 8000x magnification. Specimens form spinal cord were used to analyze proximal motor axons and glial cells surrounding motor neurons in the ventral horn.

#### Specimens from muscle

Each gastrocnemius muscle from each animal was dissected and gently stretched for 10 s before being immersed for 1 h and 30 min in the fixing solution used for perfusion. Samples were then post-fixed in buffered 1% OsO_4_ for 1 h and 30 min, dehydrated in ethanol and processed as described above. To guarantee a homogeneous analysis of the same muscle area in each mouse, we cut little blocks in the central part of the belly at the level of the wider muscle size each measuring a volume of 5 mm^3^. Since various muscle fibers orientation may lead to different structural perspectives and different measurements, each block was cut following the same longitudinal orientation. This procedure allows to keep constant the reference points and to follow the course of peripheral nerve fibers within muscle length allowing to reduce as much as possible experimental bias. Analysis at TEM was oriented by a previous light microscopy observation on 1–2 μm thick serial semi-thin sections, which were cut using a Porter Blum MT-1 or an ultramicrotome Reichert-Jung. These slices were stained with 1% toluidine blue and 1% methylene blue in 1% sodium tetraborate and they were analyzed to verify homogeneity of muscle segments and nerve fiber tracts.

Analysis of gastrocnemius muscle and peripheral nerve fibers was carried out by using TEM at 2500–6000x magnification. Muscle architecture was examined as follows: (i) intersarcomeric area, defined by the space intermingled between two sarcomers; (ii) density of mitochondria in the muscle, defined by the number of mitochondria per surface unit; (iii) percentage of altered mitochondria; (iv) mitochondrial diameter; (v) diameter of sarcoplasmic reticulum cisternae.

In peripheral nerve fibers the following parameters were analyzed: (i) density of mitochondria; (ii) percentage of altered mitochondria (defined below); (iii) mitochondrial diameter; (iv) axons owing enlarged and/or fused myelin layers.

#### Analysis of mitochondrial alterations

Mitochondria were defined as altered according to criteria being validated by previous morphological studies (Ghadially, 1988; Fornai et al., 2008a; Lenzi et al., 2012) as follows: (i) significantly decreased electron density of the matrix (dilution, vacuolization, cavitation); (ii) fragmented and ballooned cristae (intracristal swelling); (iii) partial or complete separation of the outer and inner membranes; (iv) mitochondrial swelling. This latter criterion allows to distinguish mitochondrial enlargement which might develop in physiological conditions from pathological swelling which appears as an increase in mitochondrial size due to a swelling of the mitochondrial structure.

Accordingly, the following data were calculated: (i) density of mitochondria in dendrites; (ii) density of mitochondria in the cell body; (iii) density of mitochondria in proximal and distal motor axons; (iv) percentage of altered mitochondria in dendrites, cell bodies, and motor axons; (v) mitochondrial swelling in dendrites, cell bodies and proximal and distal motor axons.

#### Additional morphometric analysis

In order to document ultrastructural alterations other than mitochondrial changes we analyzed the following: (i) dendrites diameter; (ii) ultrastructure of glial cells; (iii) clogging of motor axons. This was measured both as the percentage of clogged axonal area and percentage of motor axon with total clogging. Clogged motor axons were defined as axons filled with an electron-dense compact material in which membranes, mitochondria, and neurofilaments were no longer distinguishable but jamming in the axoplasm. Total clogging was defined by a condition in which axoplasm was no longer visible in the ultra-thin section due to the total filling with such electron-dense compact material. In some cases, the concomitant disarrangement of myelin sheet led inner myelin layers to project within axoplasm, thus contributing to axonal clogging.

In order to document the autophagy status, we analyzed double and multiple membranes vacuoles. This was done to confirm the occurrence of big stagnant autophagy vacuoles in ALS mice and their removal following lithium. These vacuoles were further identified by immunogold particles staining for LC3 and beclin-I according to the gold standard procedure to identify autophagy vacuoles.

For immuno-electron microscopy we did not apply the routine method, consisting of acrylic resins for embedding, since this procedure preserves antigens but impairs preservation of the fine morphological architecture (Lenzi et al., 2012). Thus, samples were embedded in Epon-araldite, as previously described, and incubated for 24 h at 4°C with primary antibodies anti-beclin-I (Santa Cruz Biotechnology Inc., Dallas, TX, USA, diluted 1:10) and LC3 (Santa Cruz Biotechnology Inc., diluted 1:10) in a buffer solution (PBS, 1% goat-serum, and 0.2% saponin). After washing in phosphate buffer, sections were incubated with gold-conjugated secondary antibodies (with gold particles averaging 10 nm for beclin-I and 20 nm for LC3, respectively, Sigma) diluted 1:20 in PBS for 1 h, at room temperature. This allowed co-staining for both antigens. Finally, sections were fixed with 1% glutaraldehyde and stained with uranyl acetate and lead citrate and they were examined under TEM. Control sections were not exposed to the primary antibody but they were directly incubated with the secondary antibody. This immunocytochemistry was aimed at specific recognition of autophagy vacuoles, therefore no quantitative measurement of immunogold particles was carried out.

On the other hand, immunocytochemistry for mitochondrial SOD-1 (Assay Designs, Ann Arbor, MI, USA, dilute 1:10) was carried out for quantitative purposes. SOD-1 was revealed stoichiometrically by immunogold particles (10 nm, Sigma). The count of particles localized within mitochondria was obtained from two plastic blocks randomly chosen from each experimental group. Immunogold particles were counted at TEM with a magnification of 15,000x.

#### Statistics for *Ex Vivo* studies

Values were expressed either using the absolute value or as percentage of normal numerical distributions. Data are reported as the mean or the mean percentagee ± S.E.M. This was carried out for altered mitochondria, SOD-1 immunogold particles or clogged motor axons. Inferential statistics to compare groups was carried out by using One-way analysis of variance, ANOVA, with Sheffè's *post-hoc* analysis (H_0_ probability was rejected when less than 5%, *P* ≤ 0.05).

We wish to emphasize here the need of calculating mitochondrial density (the number of mitochondria per surface unit expressed in μm^2^). In fact, the rough number of mitochondria within compartments of motor neuron may introduce experimental bias, since both disease and treatment modify motor neuron diameter (Martin et al., 2007; Fornai et al., 2008a), which in turn could bias the mitochondrial count (which could be either diluted or concentrated). Again, despite size exclusion criteria, different sizes between various compartments would not be comparable. This also applies to measurement carried out within muscle fibers. Changes in mitochondrial volume were considered separately from mitochondrial swelling which was a pure mitochondrial damage.

### *In Vitro* studies

#### Primary spinal cord cultures

Mixed primary cell cultures from spinal cords were obtained from 14-days-old mice embryos as previous described (Carriedo et al., 1996). Pregnant (14 days gestation) Swiss Webster female albino mice were purchased from Charles River Laboratories (Calco, Lecco, Italy). The spinal cords were dissected and both meninges and dorsal root ganglia were removed. The cords were then incubated for 10 min in 0.025% trypsin and cells were dissociated. The cell cultures were plated at a density of 3 “spinal cords” per well plate (35 mm dishes) on poly-D-lysine-coated glass coverslips and maintained in D-MEM supplemented with 5% FBS and 5% HS. Twenty-four hours after plating, the medium was replaced with Neurobasal supplemented with B-27 and 0.5 mM glutamine. Three days after plating cytosine arabinoside (10 μM) was added and medium was changed every 3 days. For analysis at light microscopy, cells grown on glass coverslips, within 35 mm diameter dishes, and they were transferred into cell culture plates 24 well, to perform immunohistochemistry for SMI32.

Spinal cord cultures at 8–9 days were used for toxicity experiments. The cells were administered the autophagy blocker 3-methyladenine (3-MA) 5, 10, and 20 mM for 4 h with or without lithium chloride at the dose of 0.5 mEq/L. After this time, the cultures were rapidly washed and maintained in the free medium with lithium or vehicle for 24 h. To identify motor neurons, immunocytochemistry for SMI32 was performed on primary cultures. At first, cells were fixed in a solution containing 4% paraformaldehyde in 0.1 M PBS, pH 7.3 (10 min); after washing in PBS cell cultures were incubated with Triton-X 0.1% in PB (15 min), followed by 3% hydrogen peroxide (10 min). Before incubation with the primary antibody, a blocking solution (10% normal goat serum in PBS) was added for 1 h at room temperature. The SMI32 primary antibody (mouse, 1:1000; Covance, Emeryville, CA, USA) was dissolved in PBS containing 2% normal goat serum and incubated overnight at 4°C. After rinsing in PBS the anti-mouse secondary biotinylated antibody (Vector Laboratories, Burlingame, CA USA) was used at a dilution of 1:200 for 1 h at room temperature, followed by incubation with ABC kit (1 h at room temperature, Vector Lab) and diaminobenzidine (Vector Lab). Immunostaining was analyzed at light microscopy (Nikon Eclipse 80i, Japan).

The effects of each treatment on the survival of motor neurons were measured by counting the number of SMI32 positive motor neurons in a total of 3 slides per group. All measurements were carried out at 20x magnification. Values are given as the percentage mean ± S.E.M. Comparisons between groups were made by using ANOVA with Sheffè's *post-hoc* analysis.

#### Cell lines

PC12 cells were obtained from the American Type Culture Collection and they were grown in RPMI 1640 medium supplemented with 10% heat-inactivated horse serum, 5% fetal bovine serum, penicillin (50 IU/mL), and streptomycin (50 mg/mL). Cells were seeded in 6-well plates at 1 × 10^6^ cells in a final volume of 1 ml/well and incubated at 37°C in 5% CO_2_ for 24 h.

PC12 cells were treated either with lithium chloride (1 mEq/L for 48 h) which was dissolved in culture medium (3 ml per dish) or they were added 3 ml of plain culture medium (controls). When studied for autoradiography both groups of cells received 1 μCi of [2-^3^H]adenine for 2 h, starting at 46 h after lithium/medium administration. Both lithium concentration, and the amount and time of exposure to [2-^3^H]adenine were selected based on pilot studies. [2-^3^H]Adenine (15-25 Ci mmol^−1^) was purchased from the Radiochemical Centre (Amersham, Bucks, UK). L4 photographic emulsion for electron microscopy autoradiography was obtained from Ilford (UK). In addition, plain ultrastructural morphometry (no autoradiography) was carried out from PC12 dishes which were treated with lithium or plain culture medium but they were not exposed to [2-^3^H]adenine. This was necessary to avoid that electron density of [2-^3^H]adenine impairs the fine morphometry of mitochondria and other organelles.

Cells were centrifuged at 1000 g for 5 min. After removal of the supernatant, each pellet was thoroughly rinsed in PBS. Fixation was carried out with a solution containing 2.0% paraformaldehyde/0.1% glutaraldehyde in 0.1M PBS (pH 7.4) for 90 min at 4°C. Specimens were post-fixed in 1% OsO_4_ for 1 h at 4°C, dehydrated in ethanol and embedded in Epoxy-resin. For routine TEM, ultrathin sections were contrasted with uranyl acetate and lead citrate, and examined using a Jeol JEM SX 100.

For ultrastructural morphometry, sections were examined directly at TEM at a magnification of 6000x. Each grid containing enough non-serial sections to count at least 50 cells. Several grids were observed in order to obtain a total number of at least 200 cells for each experimental group. For routine microscopy, the total number of mitochondria per cell, the number of altered mitochondria and their diameter was also measured. The altered mitochondria were described as mentioned above for *ex vivo* samples according to Ghadially (1988).

For electron microscopy autoradiography, ultra-thin sections were collected on Formvar-coated nickel grids and mounted on cork caps made adhesive with double-sided sticky tape. A monolayer of jellified Ilford L4 emulsion was then placed onto the nickel grids firmly held by the cork caps using a 2 cm wide platinum wire loop. They were then placed in black light tight boxes and exposed for up to 30 days at 4°C in a dry environment. By the end of this period, the emulsion over the grids was developed in D19 developer, fixed and thoroughly washed in distilled water.

The number and size of silver grain clusters were carefully calculated in each cell. In particular, their distribution in nuclear and cytosolic compartments was distinguished. Autoradiography grains as following: (i) number of grain clusters placed within or close to mitochondria up to 250 nm distance between the cluster contour and the closest mitochondrial outer membrane; (ii) diameter of grain clusters placed within or close (up to 250 nm distance) mitochondria (defined as previously); (iii) ratio between grain clusters placed within or close (up to 250 nm distance) to mitochondria and total grain clusters in the cytoplasm.

We counted a total of 20 cells per group. Comparisons between groups were made by using ANOVA with Sheffè's *post-hoc* analysis to compare various distances from mitochondria. Data were analyzed using Student's *t*-test for unpaired data when two groups at a single distance were compared.

**Figure 1:**
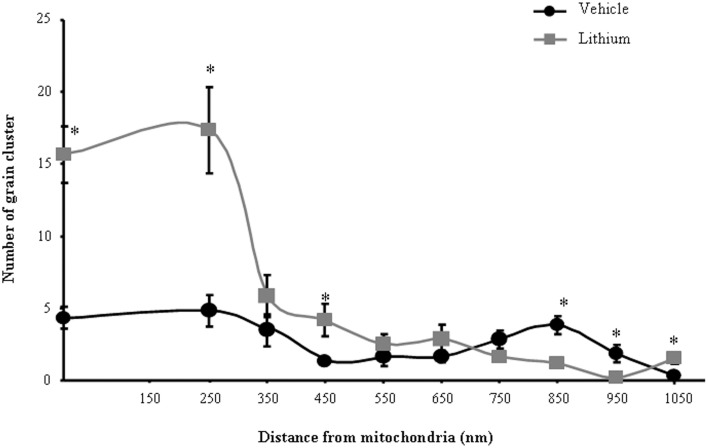
**Compartmentalization of autoradiography grain clusters**. Cytoplasmic grain clusters are counted in the presence of lithium (squares-labeled curve) or vehicle only (circles-labeled curve). Grain clusters express the presence of [2-^3^H]adenine within mitochondria (*X* = 0) and at various distances from the mitochondrial outer membrane which was expressed in nm (*X* = Nnm). The number of grain clusters for each *X*-value is reported on the Y axis. Grain clusters were counted within homogeneous areas which correspond to squares measuring 100 nm side. Under lithium administration these measurement were homogeneous within mitochondria and within an area which was distant 250 nm from the outer mitochondrial membrane. After the critical length of 250 nm, squares counted at progressive 100 nm distance possessed a number of grain clusters which decreased geometrically. Each point reports the mean ± S.E.M. of clusters. This generated a highly polarized distribution. In contrast, when cells were administered vehicle, the number of grain clusters was distributed quite homogeneously with no significant difference. This indicates that lithium produces a source of autoradiography which is centered on mitochondria according to the model published by Salpeter et al. (1978). For each point values represent the mean ± S.E.M. Comparisons between points were made by using ANOVA with Sheffè's *post-hoc* analysis. ^*^P ≤ 0.05 compared with control.

As for *ex vivo* studies, we calculated both the total number and percentage of altered mitochondria. Due to a lack of dendritic/somatic/axonal compartments in PC12 cells and homogeneous diameter of the cell line, we did not find any significant difference in providing mitochondrial data as expressed in rough number or in density. In contrast, when analyzing autoradiography data it was essential to compartmentalize morphometrical calculation of autoradiography grains based on their distance from mitochondria. In detail, the distance of 250 nm was selected as the critical length for which the highest difference was observed between lithium and vehicle-treated cells. At this distance, in lithium treated cells the number of grain clusters was the highest and it does not differ significantly from the number of grain clusters counted on the mitochondrial surface (Figure 1). We counted cytoplasmic grain clusters placed both on mitochondria and at different lengths from mitochondrial contour. This was done at progressive (100 nm) distances. Grain clusters were counted within homogeneous areas which corresponded to squares measuring 100 nm side. Under lithium administration these measurements were homogeneous within mitochondria and within an area which was distant 250 nm from the outer mitochondrial membrane. After the critical length of 250 nm, clusters which were counted within squares at progressive 100 nm distance possessed a number of grain clusters which decreased geometrically. In this way we sought to determine whether a theoretical source of autoradiography could be identified at mitochondria. In fact, assuming that [2-^3^H]adenine binds to novel synthesized mitochondrial DNA, mitochondria become the source of autoradiography diffusion according to the model provided by Salpeter et al. (1978). This assumption is based on the occurrence of authentic mitochondriogenesis under the effects of lithium. As reported in Figure 1, for lithium-treated cells, the number of autoradiography clusters starts to decrease in areas placed at a distance of more than 250 nm from mitochondrial contour. In contrast, in vehicle-treated cells there is no critical length. This demonstrates the occurrence of a polarization in the distribution of autoradiography clusters, which occurs only when lithium is present. Thus, squared area of 100 nm side ranging within mitochondria and around up to 250 nm length from mitochondria, correspond to the critical spot in which the difference in the number of autoradiography clusters reaches the maximal polarization and the maximal difference between lithium and vehicle treated cells. This starting point pertains to methodology while providing already experimental data speaking out for *in situ* evidence of mitochondriogenesis induced by lithium. The occurrence of nuclear staining was clearly independent from mitochondria (ranging way in excess of 250 nm and it was bound to nuclear DNA). Of course this staining does occur but it does not interfere with the count in the cytosol where the staining was really scarce at distance from mitochondria. Thus, two sources of radioactive staining occur: (i) nuclear chromatin; (ii) cytosolic mitochondria. Nuclear and cytosolic compartments are generally separated one from each other by large areas of faint randomly placed staining, which is similar to the loss of radioactivity occurring in the cytosol for areas placed between mitochondria. When a mitochondrion was placed at a distance not exceeding 250 nm from the nucleus (less than 10% of mitochondria) this was not included in the count. This was done to avoid bias of contaminating counts with the nuclear source of clusters. The cluster density we counted within mitochondria corresponds to that measured within a 250 nm range from mitochondrial contour. This explains graph in Figure 1 where the *Y*-values of radioactive clusters was constant in the interval between *X* = 0 (corresponding to the mitochondrial surface) and *X* = 250 nm. When counts were carried out in space intervals extending above 250 nm, at 100 nm progressive intervals we measured a geometric fall in the number of clusters (Figure 1). This curve of cluster distribution produced by lithium was abolished in vehicle-treated cells.

## Results

### Morphometry of mitochondria within cell bodies of motor neurons, lithium–and disease-modifying effects

**Figure 2:**
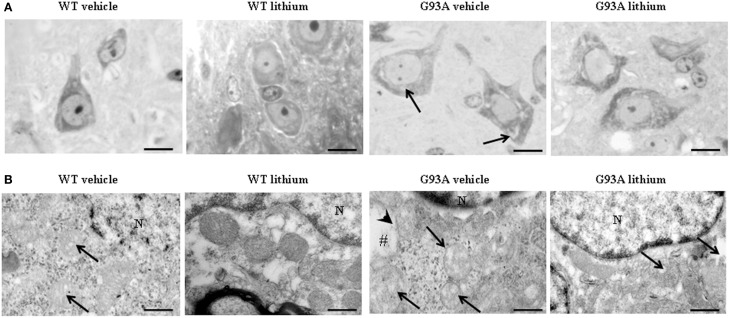
**Representative pictures at light microscopy and transmission electron microscopy of motor neurons. (A)** Semi-thin sections are obtained from lamina IX region of the anterior horn of mice spinal cord stained with methylene and toluidine blue. In WT and G93A mice motor neurons appear as multi-polar cells, where the nucleus is not condensed and the nucleolus is well-evident. Motor neurons were chosen also according to a size exclusion criterion as reported in the Materials and Methods. The size of motor neurons increases in G93A mice administered vehicle. In this group the cytoplasm is filled with large vacuoles (arrows). Following lithium administration, large vacuoles disappear. **(B)** Motor neurons in the anterior horn were analyzed for TEM ultrastructural morphometry of mitochondria. Mitochondria from WT mice treated with vehicle are regularly shaped with homogeneous matrix. In this group only a few mitochondria possess slightly ballooned cristae (arrows). This architecture is further improved by lithium administration, which increases electron-density of the matrix and the integrity of the cristae. In G93A mice administered vehicle, mitochondria exhibit a partial (arrows) or total (#) matrix dilution and dramatic fragmentation of the cristae (arrowhead). Remarkably, in G93A mice lithium reverses mitochondrial alterations leaving only a few altered mitochondria (arrows). N, nucleus. Scale bars = **(A)** = 30 μm; (WT vehicle) = 0.46 μm; (WT lithium) = 0.30 μm; (G93A vehicle) = 0.60 μm; (G93A lithium) = 0.5 μm.

**Figure 3:**
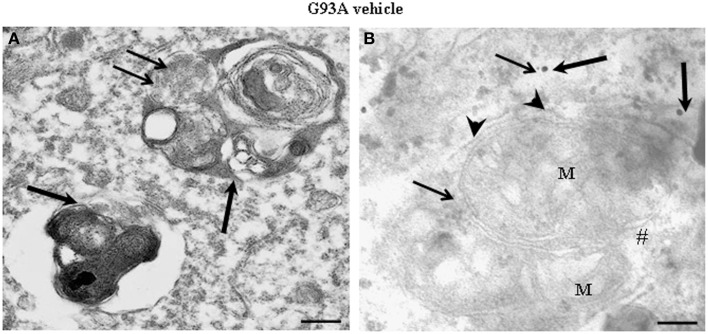
**Representative electron micrographs from G93A mice**. These pictures show at high magnification motor neurons from vehicle-treated G93A mice. These neurons possess a cytoplasm filled with large, stagnant autophagy vacuoles (thick arrows). Vestigial mitochondrial cristae (thin arrows) are present in a multiple-membranes autophagy vacuole **(A)**. Two degenerated mitochondria, wrapped by a close autophagy vacuole (arrowheads), show ballooned cristae, and breakings of outer and inner membranes (#). LC3 (thick arrows) and beclin-I (thin arrows) immunogold particles are closely associated with autophagy membranes thus witnessing as the established gold standard to identify autophagy vacuoles **(B)**. M, mitochondria. Scale bars = **(A)** = 0.53 μm; **(B)** = 0.39 μm.

**Figure 4:**
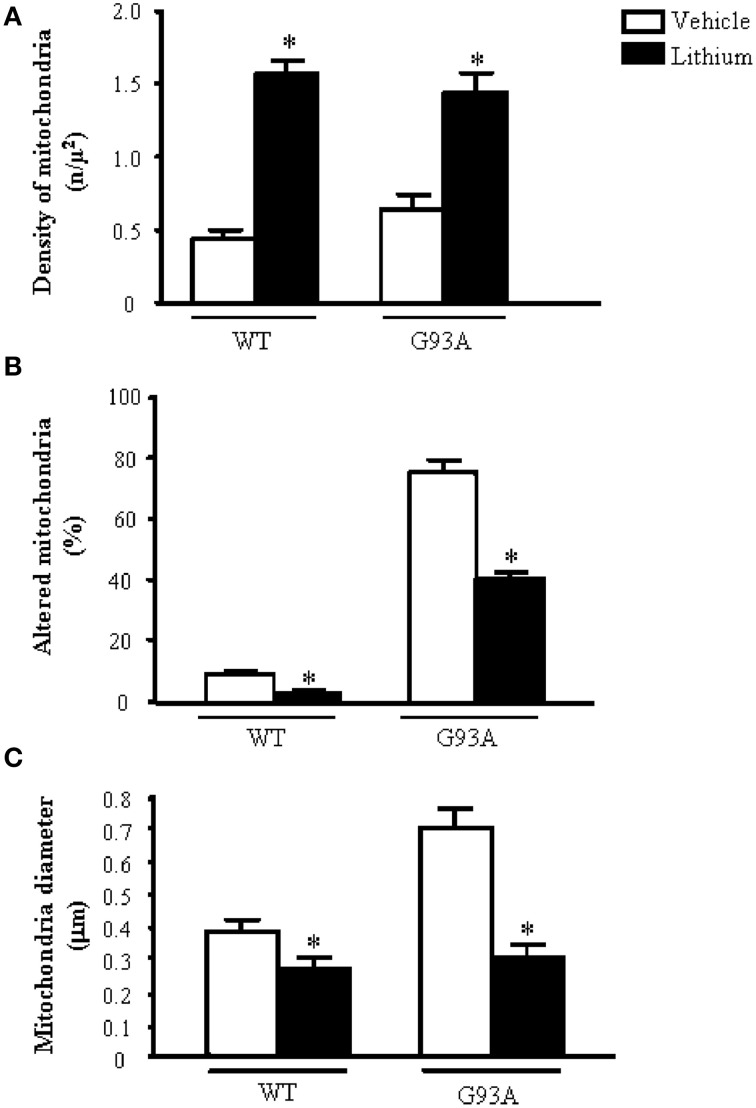
**Ultrastructural morphometry of mitochondria within cell body of motor neurons**. The soma (cell body) of motor neurons from the anterior horn of the mouse spinal cord was counted to establish the density **(A)** the alterations **(B)** and the size **(C)** of mitochondria. The criteria used to define a mitochondrion as altered were extensively reported in the Materials and Methods Section. Mitochondrial density within cell body is dramatically augmented by lithium administration both in WT and G93A mice **(A)**. In line with the preservation of mitochondrial architecture observed in Figure 2B, lithium significantly reduces the percentage of altered mitochondria **(B)**. As noticed from representative pictures a re-shaping effect of lithium is also counted The size of mitochondria was increased in G93A mice administered vehicle but it is brought back to WT values when G93A mice received lithium. Remarkably, such re-sizing effects of lithium also occur in WT mice, where lithium further decreases mitochondrial diameter in WT mice **(C)**. Values are given as the mean ± S.E.M. Comparisons between groups was made by using One-way ANOVA. ^*^P ≤ 0.05 compared with vehicle-treated mice in graph.

Figure 2A shows representative semi-thin sections from the anterior horn of WT and G93A mice treated either with vehicle or lithium. It is evident the general shape of motor neurons which appear as multipolar cells, in which nucleus is not condensed and the nucleolus is well evident. The size of motor neurons corresponds to the size exclusion criterion. Nonetheless, in these representative pictures we reported the disease-dependent increase in motor neuron size (compare G93A vehicle with WT vehicle) and the effects of lithium, which brings back motor neuron diameter to control values (compare G93A lithium with WT vehicle). Interestingly, as we already published (Fornai et al., 2008a), the size of motor neurons in WT is further decreased by the effects of lithium. This size change does not exceed 10% of diameter and it does not affect significantly the size exclusion criterion. In addition, within motor neurons of G93A mice a prominent vacuolization is evident which is markedly reduced by lithium administration (Figure 2A). These semi-thin sections addressed further studies at electron microscopy reported in representative Figure 2B showing well-shaped and regularly-sized mitochondria in WT mice compared with swollen mitochondria of G93A mice, which possess matrix dilution and breaking of the cristae. This is further documented in Figure 3 showing swollen mitochondria with fragmented and swollen cristae with a rupture of both inner and outer membranes. Remarkably, mitochondria from ALS mice are abnormally swollen up to an average diameter which is way in excess to what it was measured in WT mice (0.69 ± 0.05 and 0.38 ± 0.025 respectively, Figure 4). These altered mitochondria often appear within autophagy vacuoles which are identified by the gold standard procedure according to Klionsky et al. (2012) as TEM-identified double membrane organelles (Figure 3A), staining both for LC3 and beclin I (Figure 3B). In these mice, the amount of giant/altered mitochondria, which was found within big stagnant autophagy vacuoles, was very high (percentage of 75.25 ± 3.1 %, Figure 4). As shown in Figure 2B, the mitochondrial alterations were reduced by lithium, thus providing a gold standard TEM validation for lithium-induced autophagy. In detail, as counted in graphs of Figure 4 lithium increases the density of mitochondria (Figure 4A), it reduces the amount of altered mitochondria (Figure 4B), and it reduces mitochondrial diameter (both in normal and swollen mitochondria, Figure 4C). Thus, in ALS mice administered lithium for about 170/180 days we found a marked suppression of giant swollen mitochondria with slighter matrix dilution and rare cristae fragmentation. These mitochondria were barely detectable within autophagy vacuoles, which in turn did not appear as big stagnant vacuoles but they were similar to controls.

### Morphometry of mitochondria within motor neuron dendrites, lithium–and disease-modifying effects

**Figure 5:**
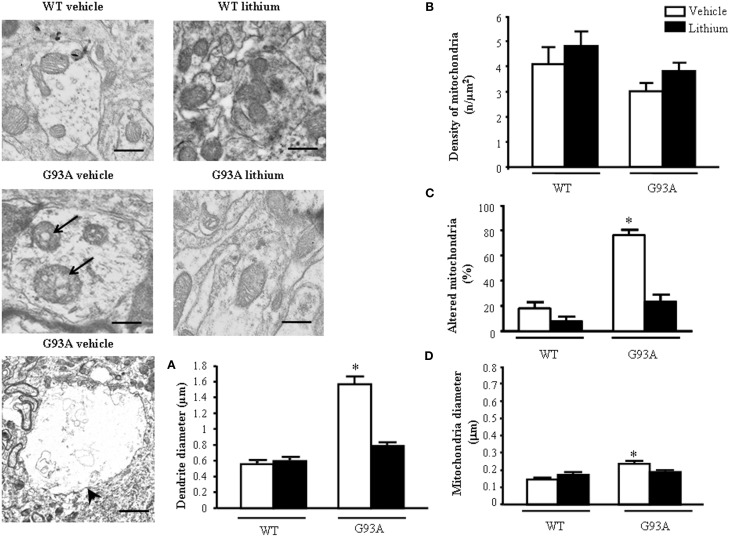
**Ultrastructural morphometry of mitochondria within dendrites of motor neurons**. Mitochondria within dendrites of motor neurons of the anterior horn of mice spinal cords are representatively reported. Mitochondria from dendrites of vehicle-treated G93A mice possessed swollen cristae (arrows) which do not appear neither in WT nor in lithium-treated G93A mice. Low magnification representative picture from vehicle-treated G93A mice shows giant dendrites (arrowhead) compared with WT mice, this is fully reversed by lithium as counted in graph **(A)**. Graph **(B)** reports a slight non-significant reduction of mitochondrial density in vehicle-treated G93A mice compared with WT, which was not affected by lithium. In contrast, altered mitochondria were significantly increased in vehicle-treated G93A mice compared with WT mice, although lithium treatments restore the percentages of altered mitochondria in G93A to values similar to WT mice **(C)**. The mitochondrial diameter in dendrites from G93A mice was slightly but significantly increased following vehicle, but it was normal following lithium **(D)**. Values are the mean ± S.E.M. Comparisons between groups were made by using One-way ANOVA. ^*^P ≤ 0.05 compared with other groups in graph. Scale bars: WT vehicle and WT lithium = 0.3 μm, G93A vehicle and G93A lithium = 0.4 μm, G93A vehicle (low magnification) = 1.3 μm.

Dendrites were partly reminiscent of what we already described within the neuron cell body (Figure 5). However, a striking effect concerns dendritic volume. G93A mice possess giant dendrites compared with WT mice as evident in representative pictures and in graph of Figure 5A.This effect is way more pronounced of what already described for the cell body of motor neurons. This explains why, despite an increase in the total number of mitochondria the density of these organelles was attenuated in the dendrites of G93A mice (Figure 5B). In detail, mitochondrial morphology was deranged in G93A mice compared with WT. These effects were prevented by lithium, which produces a mitochondrial morphology similar to WT both concerning the architecture and diameter of dendritic mitochondria (Figures 5C,D). Lithium further augmented mitochondrial number but this effect was mitigated by the measurement of mitochondrial density (which is in line with the giant dendritic volume of G93A mice). When lithium was administered to WT mice an improvement of mitochondrial architecture and increase in mitochondrial density were observed (Figure 5). The percentage of altered mitochondria, which was significantly higher in G93A mice administered vehicle, was brought back to WT values following lithium administration (Figure 5C).

### Morphometry of mitochondria within proximal motor axons, lithium–and disease-modifying effects

**Figure 6:**
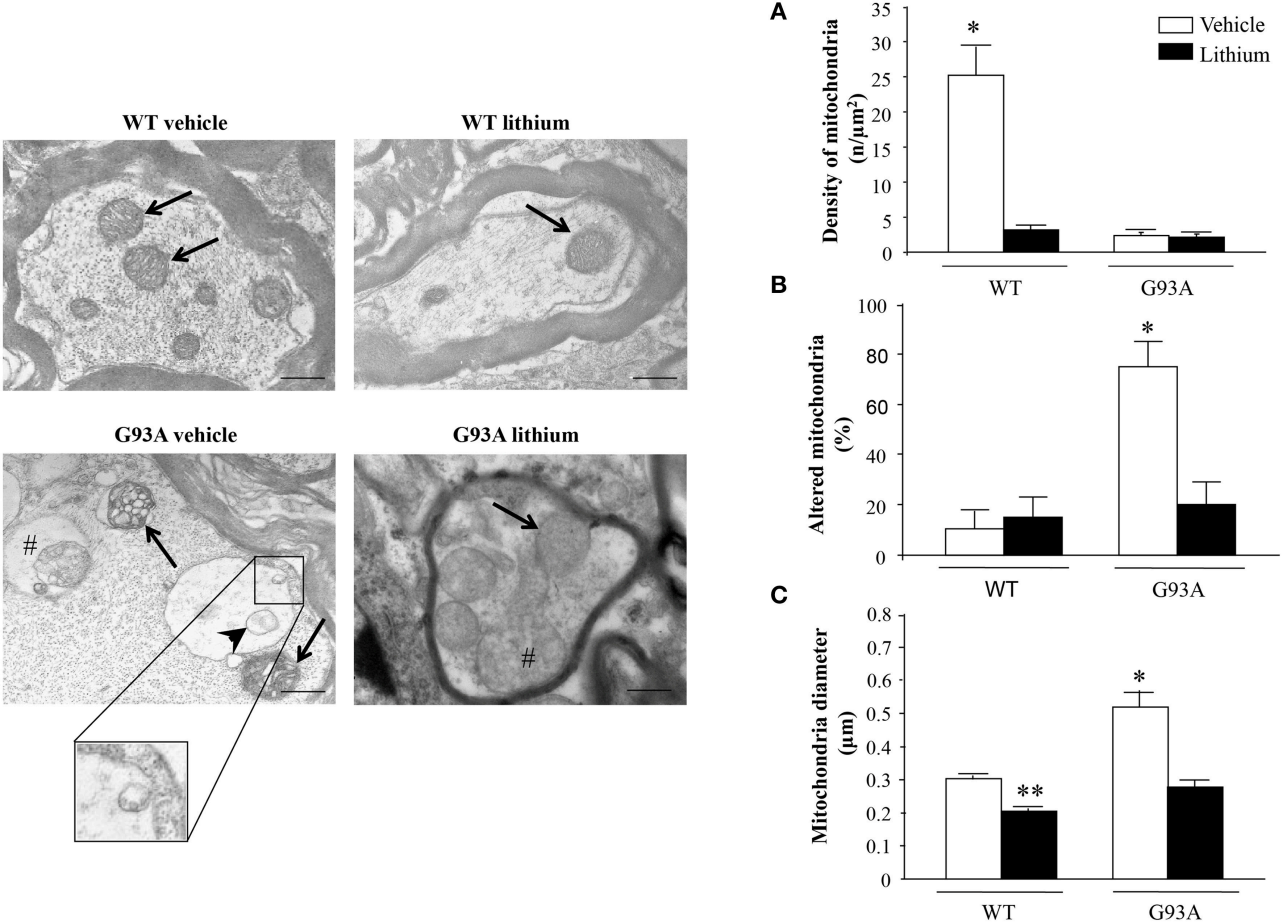
**Ultrastructural morphometry of mitochondria within proximal axons of motor neurons**. Mitochondria within motor axons from the anterior horn are reported in representative pictures. As it appears in representative pictures mitochondria within motor axons of WT mice treated either with vehicle or lithium possess well-conformed mitochondria (arrows). In contrast, motor axons from a vehicle-treated G93A mouse possesses dramatic mitochondrial alterations surpassing those representatively described within the cell body in Figure 2B. These mitochondria possess marked enlargement of the cristae (arrows) and a total derangement and vacuolization of a mitochondrion (arrowhead). The axonal internal structure shows large vacuoles (#) containing a mitochondrion. Note the vestigial cristae within vacuolated mitochondrial inner structures (insert). Lithium treatment reduces the severe alterations in axons showing well-conformed mitochondria (arrow) with some spots of swollen cristae (#). In graph **(A)** mitochondrial density is markedly reduced in all groups but in WT mice administered vehicle. The percentage of altered mitochondria **(B)** and mean mitochondrial diameter **(C)** which were significantly increased in G93A mice administered vehicle were reduced by lithium to values compared with controls. Values are the mean ± S.E.M. Comparisons between groups were made by using One-way ANOVA. ^*^P ≤ 0.05 compared with others groups. ^**^P ≤ 0.05 compared with vehicle-treated WT. Scale bar = 800 nm.

When observing representative pictures of motor axons, in G93A mice (Figure 6) dramatic mitochondrial alterations were evident which surpassed those described for mitochondria in the cell body and dendrites of motor neurons. Severe architectural alterations of mitochondria were fairly prevented by lithium (Figure 6).

Unexpectedly, while lithium increases the density of mitochondria in the cell body (Figure 4A) this does not occur within proximal axons where the density of mitochondria in G93A and WT mice treated with lithium was reduced similarly to G93A mice treated with vehicle (Figure 6A). Thus, within proximal axons lithium and the disease condition independently produce a similar mitochondria reduction (Figure 6A). However, altered mitochondria were markedly suppressed by lithium administration of G93A mice (Figure 6B). The percentage of altered mitochondria in the proximal axon was similar to that counted in the cell body. This is corroborated by representative pictures, which demonstrate mitochondrial impairment in proximal motor axons (see insert of Figure 6). However, in the proximal axon there is a significant decrease in the total number of mitochondria which does not occur in the cell body. This suggests that disease progression leads to a similar mitochondrial damage in the cell body and proximal axons but the number of mitochondria is frankly diminished only in the proximal axon. Interestingly, this effect was observed also in G93A mice administered lithium and, most remarkably it was measured in lithium-administered WT mice. One might argue that this count is explained by the lack of mitochondrial replacement (likely sustained by mitochondriogenesis) within proximal axons both in disease conditions as well as under accelerated mitophagy promoted by lithium. Noteworthy, the few mitochondria counted both in G93A and WT mice treated with lithium appear to be structurally preserved (representative pictures of Figure 6). This is confirmed by counts of altered mitochondria and mean mitochondrial diameter within motor axons (Figures 6B,C). Moreover, one should consider that, when counting mitochondria at this proximal level within the cord or in the ventral root as carried in this paragraph and in the work by Parone et al. (2013), this does not necessarily reflect the mitochondrial status at distal axons where the critical damage occurs. This is the first time, which a separate count for axonal mitochondria, was carried out to distinguish what occurs in the proximal axons compared with peripheral axon which is reported in the next paragraph.

### Morphometry of mitochondria within distal axons, lithium–and disease-modifying effects

**Figure 7:**
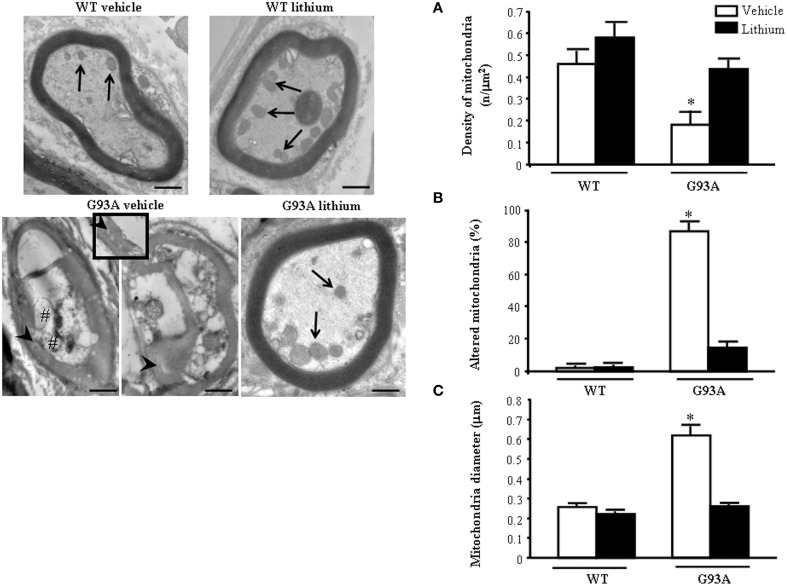
**Representative pictures and morphometry of distal axons**. Electron micrographs of cross-sections of myelinated axons. Vehicle- and lithium-treated WT mice and lithium-treated G93A mice show well-organized myelin sheaths surrounding an axoplasm with normal neurofilaments and well healthy mitochondria (arrows). In vehicle-treated G93A mice abnormal myelin sheaths, including disruption and disordered arrangement of fibers, are visible. Arrowheads point to a particular zone of disrupted myelin sheaths with loss of stratification (see also insert). Mitochondrial vestigia (#) are also observed in the altered axoplasm of G93A vehicle mice. Graph **(A)** shows a significant reduction of mitochondrial density in vehicle-treated G93A mice compared with other groups. In addition, the percentage of altered mitochondria **(B)** and the mitochondrial diameter **(C)** are significantly increased in G93A vehicle mice. Both effects were prevented by lithium administration. Values are the mean ± S.E.M. Comparisons between groups were made by using One-way ANOVA. ^*^P ≤ 0.05 compared with other groups. Scale bars: WT vehicle, WT lithium, G93A vehicle = 0.7 μm, G93A lithium = 0.6 μm, insert = 0.2 μm.

The morphometry of distal axons reveals remarkable differences compared with proximal axons. As shown in representative pictures of Figure 7 the mitochondrial damage is further worsened in vehicle-administered G93A mice compared with every other compartment including proximal axons.

The axoplasm of distal axons is entirely jammed by abnormal structures where normal constituents are no longer distinguishable (see next paragraph). These abnormal structures include the myelin sheet, which seems to intrude the axoplasm itself and is altered (see insert). This is reverted by lithium. In fact in the peripheral axons the loss of mitochondria is prevented by lithium which increases mitochondrial number similarly to WT mice (Figure 7A) Lithium also provides significant protection against mitochondrial damage which is mostly evident in G93A mice administered vehicle (Figure 7B). The amount of mitochondrial damage measured in the distal axons involves almost the total number (more than 90%). This implies that preserved mitochondria in the distal axons are more than two-fold the preserved mitochondria within proximal axons, where only a subtotal (less than 80%) mitochondrial damage takes place (compare Figure 7B with Figure 6B).

These data fully confirm the occurrence of the worst mitochondrial damage in peripheral axons as described also in ALS patients (Shi et al., 2010).

These data indicate the need to extend the ultrastructural morphology of ALS axons toward the peripheral segments to unravel the significance of axonal compared with perikaryal alterations. This is reciprocated by the occurrence of protective effects of lithium both on mitochondrial density (Figure 7A), and mitochondrial alterations (Figure 7B). At this level, lithium reduced mitochondrial diameter due to a specific effect on preventing mitochondrial swelling (Figure 7C).

### Extra-mitochondrial morphometry of axons, lithium–and disease-modifying effects

**Figure 8:**
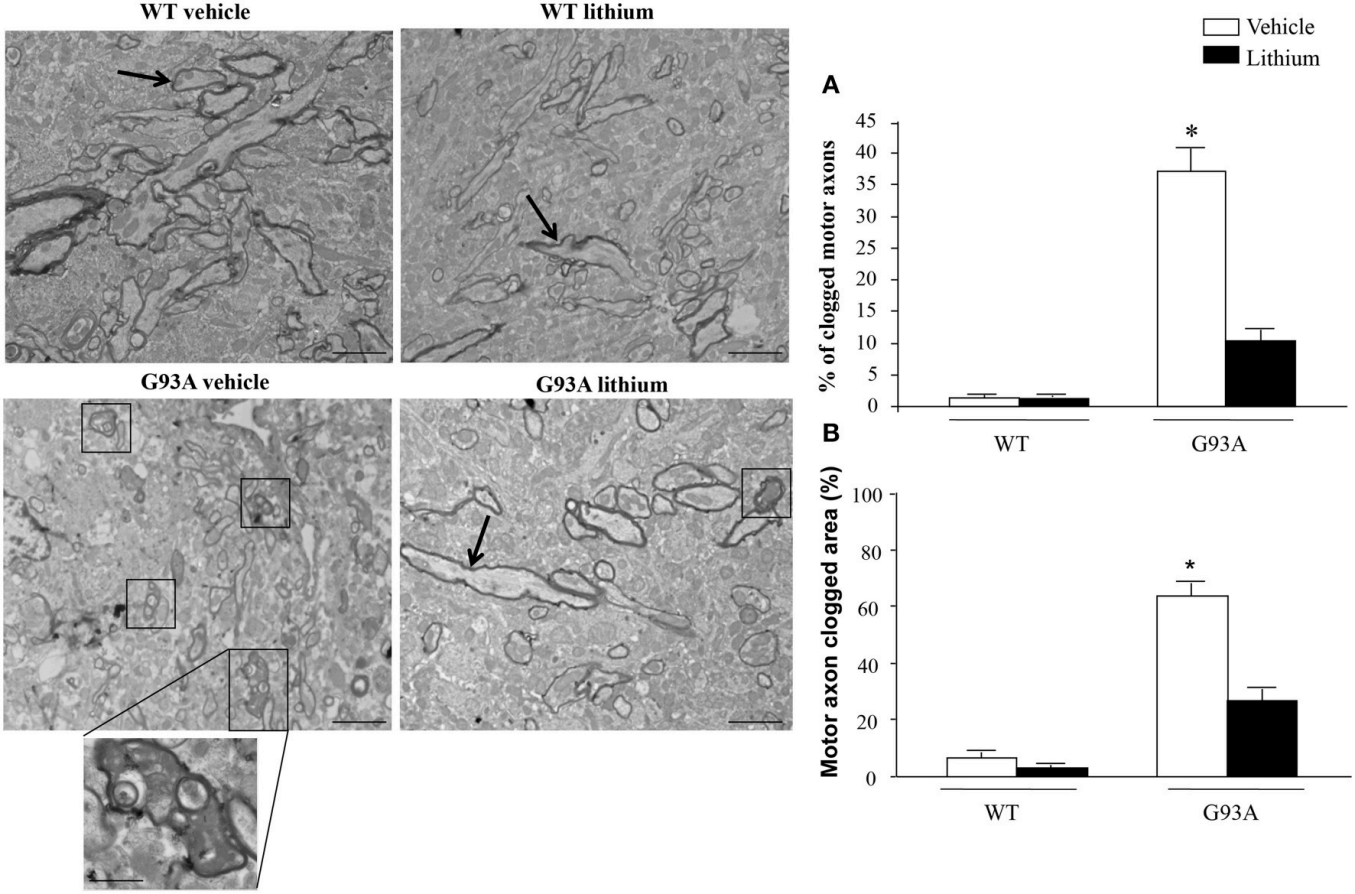
**Extra-mitochondrial motor axon alterations**. WT vehicle- and WT lithium-treated mice show well-conformed myelinated axons (arrows). No obstructive material is observed within the axoplasm. In contrast, in G93A vehicle-treated mice, axons show an axoplasm filled with an electron-dense, heterogeneous, condensed aggregated structures clogging the axonal lumen (see detail at high magnification). Lithium treatment reduces the percentage of clogged motor axons (square), as showed in graph **(A)**. Graph **(B)** shows the percentage of clogged area within motor axon surface, which appears significantly increased in vehicle-treated G93A mice, this effect is significantly reduced by lithium treatment. Values are the mean ± S.E.M. Comparisons between groups were made by using One-way ANOVA. ^*^P ≤ 0.05 compared with other groups. Scale bars: WT vehicle and G93A lithium = 1.5 μm, WT lithium and G93A vehicle = 1.8 μm. G93A vehicle (high magnification) = 0.5 μm.

When observing the extra-mitochondrial alterations within motor axons (Figure 8) low magnification provides a clear perspective of axonal clogging which was further detailed in previous Figure 7 by abnormal structures, which were jamming the axoplasm. In G93A mice administered vehicle this effect leads to electron-dense material which fills almost totally the axoplasm, which is cleared back significantly by lithium as shown by representative pictures and graphs of Figure 8. These jams are clearly visible in the insert at high magnification of Figure 8 in vehicle-administered G93A mice as mixed structures clogging the axoplasm. These axonal inclusions are frankly pathological as witnessed by their amorphous structure and their total absence in axons from WT mice (Figure 8A). This contrasts with the occurrence of a critical clogging (more than 60%) of axoplasm which was present in several motor axons from G93A mice (Figures 8A,B), while it was limited to 26% of the luminal area of a few motor axons in G93A mice administered lithium (Figures 8A,B). This material we described here selectively in the motor axon compartment is likely to produce significant deleterious effects concerning axonal physiology at least affecting axonal transport which indeed is functionally impaired (Magrané et al., 2012, 2014). In fact, the amount of clogged area almost reaches 70% of the axonal area measured in 2D electron microscopy. The administration of lithium produces a remarkable effect since it tears down the clogged area of more than two-fold from 63.58 to 26.56%, which means a net gain of axonal lumen of more than 50% (Figure 8B). Incidentally, the clearance induced by lithium of more than two-fold the clogging of motor axons is similar to the increase in mitochondrial density and the amount of spared mitochondria (more than two-fold), which was measured in the distal axons. This indicates that by increasing the available axonal lumen is associated to the increase of novel mitochondria transported downstream and the increased number of healthy mitochondria counted at distal level.

### Structure of muscles, lithium–and disease-modifying effects

**Figure 9:**
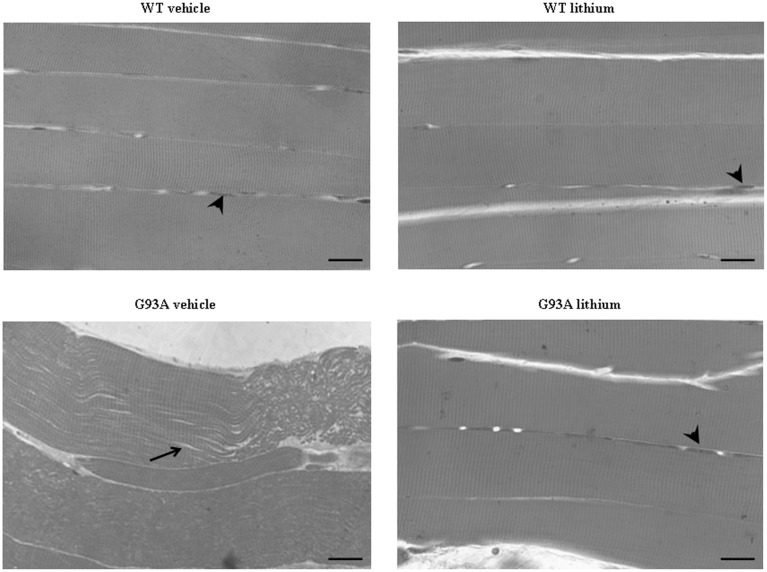
**Representative pictures at light microscopy of semi-thin sections from gastrocnemius muscle**. Semi-thin sections were stained with methylene and toluidine blue. Vehicle- and lithium-treated WT mice and lithium-treated G93A mice exhibit longitudinal, regular, parallel arrangement of aligned muscle fibers showing the typical banding pattern. Nuclei at the periphery of the fibers are visible (arrowheads). Muscle from vehicle-treated G93A mice shows unparalleled and disarranged fibers and myofibrils. In this latter group the space among fibers appears enlarged (arrow). Scale bar: 28 μm.

In semi-thin sections from the gastrocnemius muscle (Figure 9) we could dissect the homogeneous segment to be analyzed under electron microscopy. The severe disarrangement of muscle structure in vehicle-administered G93A mice is clearly evident (Figure 9). Similarly, the re-alignment and the lack of abnormal architecture of muscle fibers can be appreciated in Figure 9 showing G93A mice administered lithium.

**Figure 10:**
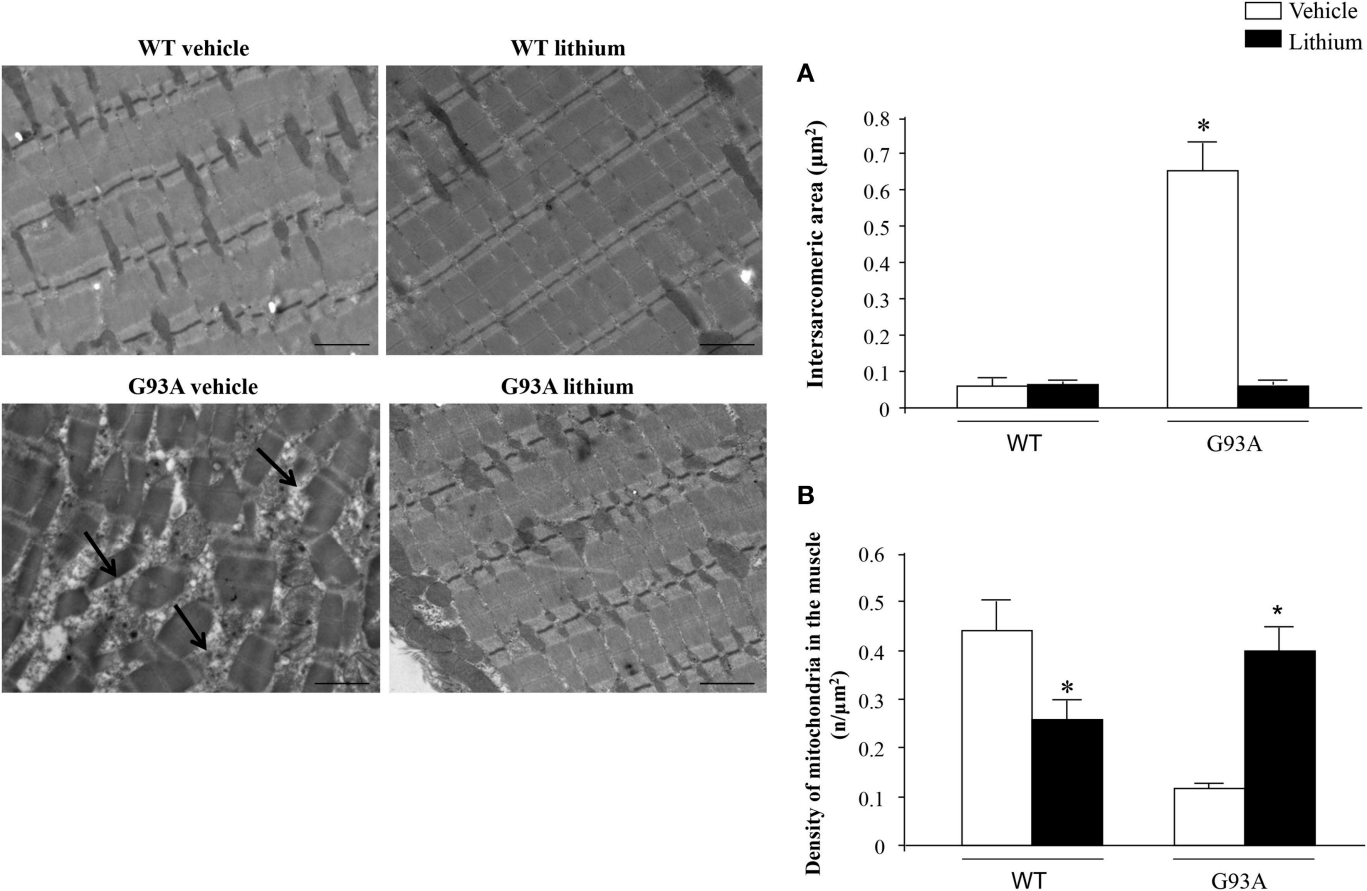
**Representative pictures and ultrastructural morphometry from gastrocnemius muscle**. Vehicle- and lithium-treated WT mice and lithium-treated G93A mice show well-arranged mitochondria and myofibrils with the typical pattern of dark and light bands. No ultrastructural alterations were detected in any of these groups. It is worth of noting that the image from WT lithium-treated mice possess a surprisingly geometrical alignment of sarcomers. Muscle from vehicle-treated G93A mice shows several disarranged areas with a significant increase in intersarcomeric area (arrows and graph **A**) containing a few mitochondria (graph **B**), these effects being greatly reversed by lithium. Values are the mean ± S.E.M. Comparisons between groups were made by using One-way ANOVA. ^*^P ≤ 0.05 compared with other groups. Scale bar = 1 μm.

**Figure 11:**
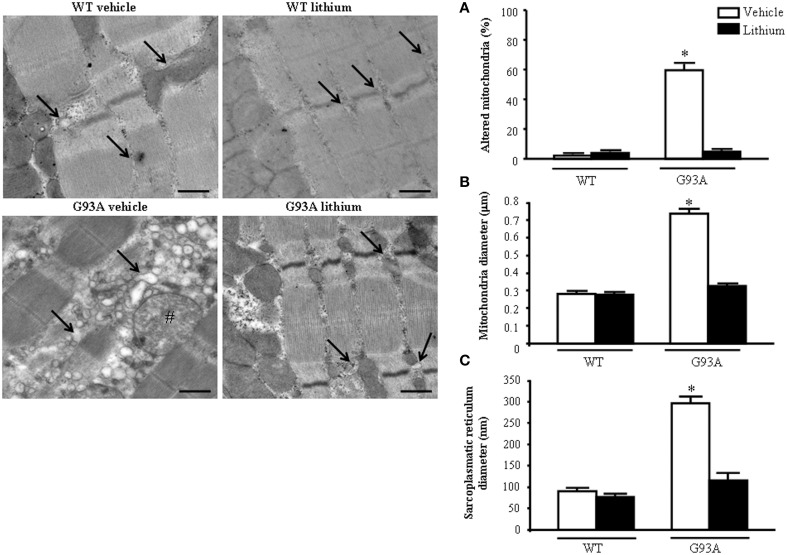
**Representative pictures at high magnification and morphometry of mitochondria and sarcoplasmic reticulum from gastrocnemius muscle**. Vehicle- and lithium-treated WT mice show a normal pattern of mitochondria and sarcoplasmic reticulum (arrows). Vehicle-treated G93A mice show altered swollen mitochondria (#) with enlarged cristae and sarcoplasmic reticulum with swollen cisternae (arrows); lithium treatment markedly prevents the occurrence of these ultrastructural alterations **(A–C)**. Values are the mean ± S.E.M. Comparisons between groups were made by using One-way ANOVA. ^*^P ≤ 0.05 compared with other groups. Scale bar: 0.4 μm.

When examined at TEM, representative pictures of Figure 10 show that, within muscle fibers from saline-administered G93A mice, there was a severe loss of the normal sarcomeric structure as well as the regular alignment of sarcomers. These muscle fibers from vehicle-administered G93A mice could no longer be recognized as composed of sarcomeric units. The severe disarrangement which appears in G93A mice was measured by counting the distance between vestigial sarcomers (Figure 10A) which was increased more than ten-fold compared with WT. In these muscle fibers the effects of lithium were most remarkable since the intersarcomeric area of lithium-administered G93A mice was re-instated to values similar to WT. This is well evident also from representative pictures cited from Figure 10, showing that lithium substantially preserves the sarcomeric architecture in the muscle fibers. The fine alignment of sarcomers was even more geometrically correct when WT mice were administered lithium, but this pertains only to the appreciation of the pictures and it was not specifically measured. It is likely that, the density of mitochondria under the effects of lithium might contribute to this effect. In fact, when counting the number of mitochondria in G93A mice a massive decrease was measured, which was significantly prevented by lithium (Figure 10B). Interestingly, in WT mice there was a non-significant trend toward a decrease in the number of mitochondria. In any case dramatic mitochondrial alterations in the muscle of G93A mice were totally abolished by lithium administration as can be appreciated in representative pictures of Figure 11 and as reported by the counts of graph in Figure 11A. This effect was also evident by measuring the mitochondrial swelling in each group which was graphed in Figure 11B as an increase in mitochondrial diameter. Remarkably, the swelling of sub-cellular structures in the muscle of G93A mice extends to the sarcoplasmic reticulum which was increased three-fold compared with wild types (representative pictures of Figure 11 and graph of Figure 11C). Again, the administration of lithium abolished such an alteration. Thus, the preservation of various components of the gastrocnemius muscle structure was remarkable under the effects of lithium. This is key, since the loss of muscle structure and architectural disruption which occurs otherwise in vehicle-administered G93A mice along with classic mitochondrial alterations were really impressive.

### *In Vitro* study on cell lines and primary cultures from spinal cord

In order to better document the effects observed *in vivo* on mitochondrial morphology and number we measured the effects of lithium in a PC12 cell line which owns much less experimental variables. We further extended the mechanistic analysis on the role of lithium and autophagy in a primary cell cultures from spinal cord.

**Figure 12:**
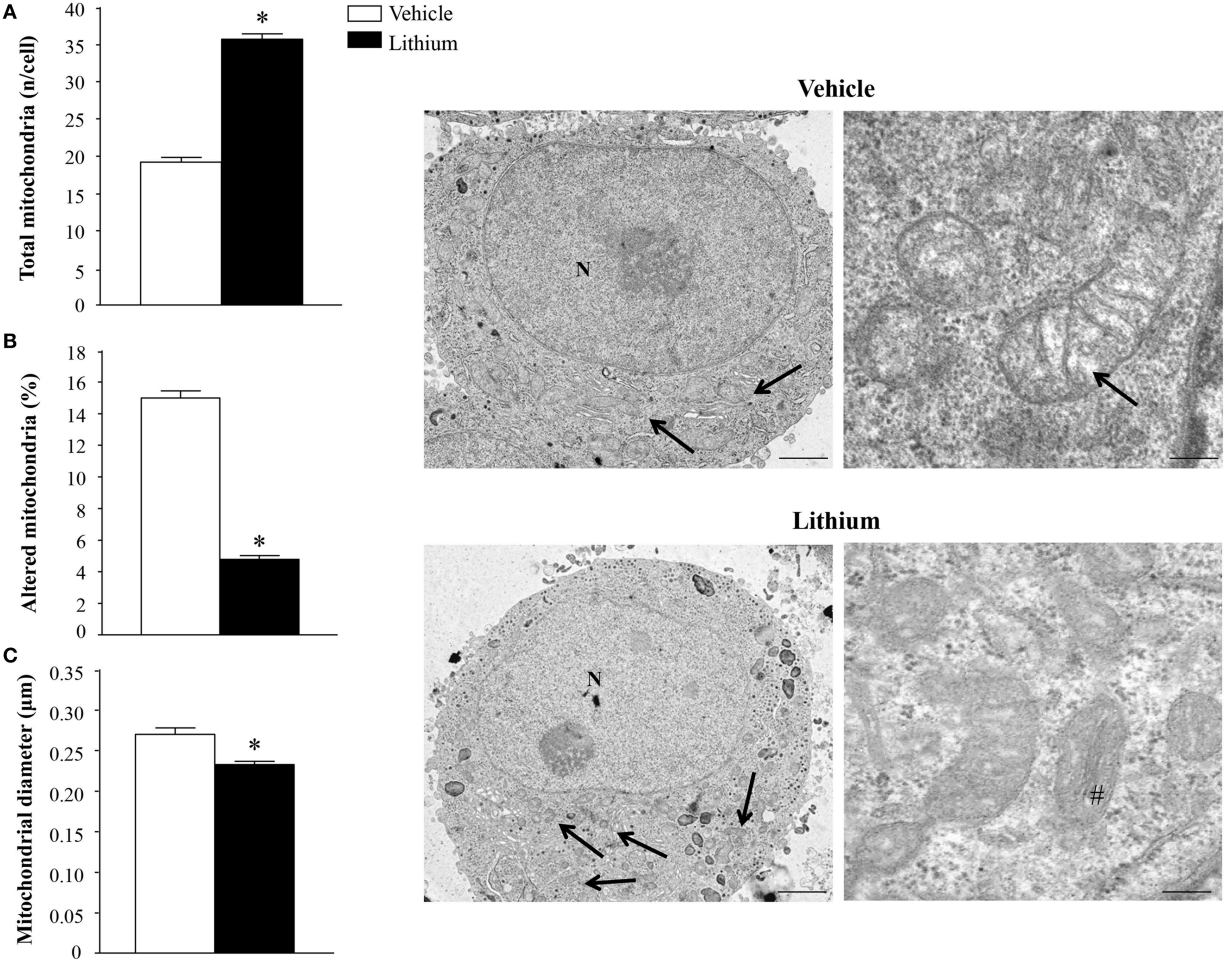
**Ultrastructural morphometry of mitochondria ***in vitro*** in PC12 cells**. Lithium exposure *per se* significantly increases the total number of mitochondria **(A)** and reduces the percentage of altered mitochondria **(B)**. Again, lithium reduces mitochondrial diameter **(C)**. Representative TEM pictures show the dramatic increase in the number of mitochondria after lithium exposure, which is already evident at low magnification (arrows). In a higher magnification of control PC12 cell a slight mitochondrial matrix dilution (arrow) is observed; this alteration is no longer present after lithium administration (#). Values are the mean ± S.E.M. Comparisons between groups were made by using Student's *t*-test for unpaired data. ^*^P ≤ 0.05 compared with controls. Scale bars: (low magnification) = 1.45 μm; (high magnification) = 0.14 μm.

As shown *ex vivo* in the spinal cord, in PC12 line lithium increases two- fold the number of mitochondria compared with vehicle (Figure 12A). In this cell line, which owns aberrancies inherent with their shift from a rat pheocromocytoma (Fornai et al., 2007) the number of mitochondria, which eventually undergo spontaneous alterations is noticeable and reaches 15% of total mitochondria (Figure 12B). These spontaneous mitochondrial degeneration was suppressed by lithium administration (which reduced three-fold the percentage of altered mitochondria, Figure 12B). Thus, considering that lithium increases two-fold the total number of mitochondria, while it decreasing three-fold the percentage of altered mitochondria, the total number of mitochondria which are structurally preserved by lithium corresponds roughly to six-fold the number of preserved mitochondria measured in control. At the same time, lithium reduces significantly the mitochondrial diameter (Figure 12C). This size reduction was counted independently from mitochondrial swelling but it was counted considering only healthy mitochondria. This effect suggests the occurrence of mitochondriogenesis which is known to produce small densely packed mitochondria with a well-marked architecture both concerning arrangement of the cristae and matrix electrondensity. In fact, when observed in representative TEM pictures (Figure 12), lithium-treated cells consistently show a dramatic increase in mitochondrial density, which clearly appears at first glance (see lower magnification in the left row of Figure 12). At the same time, at higher magnification (in the right row of Figure 12), the mitochondrial architecture was well-delineated in lithium-treated cells. These results confirm the hypothesis that lithium possesses mitochondriogenetic effects.

**Figure 13:**
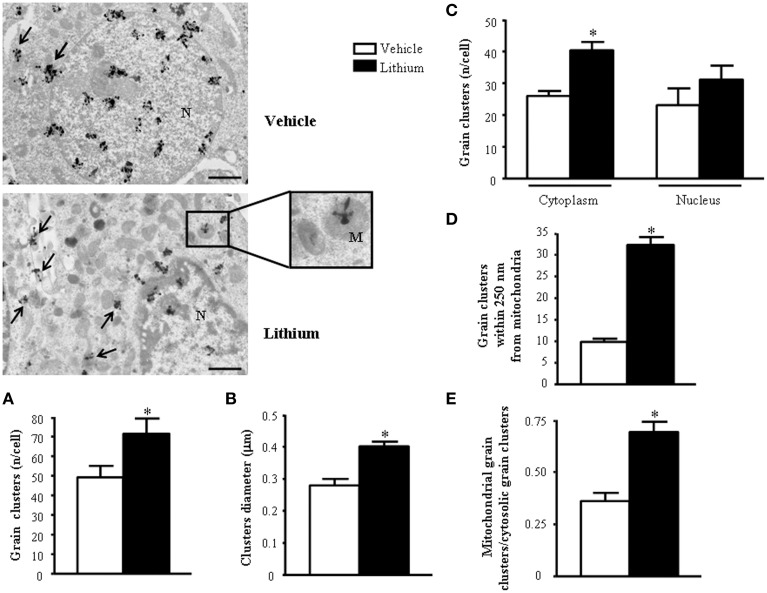
**Representative TEM autoradiographs on distribution of [2-^3^H]adenine**. TEM autoradiographs of PC12 cells exposed to [2-^3^H]adenine for 2 h. Grain clusters of [2-^3^H]adenine are distributed within nucleus and cytoplasm (arrows) of both control and lithium-treated cells. In detail, following lithium administration grain clusters increase in number **(A)** and diameter **(B)** and polarize in the cytoplasm (closer to mitochondria [insert]) **(C)**. To fully consider these data, the reader is suggested to go back to Figure 1, which demonstrates the significant compartmentalization of grain clusters in the cytosol. In detail, while the total number of grain clusters in the cells is slightly increased by lithium **(A)**. Such an increase is more pronounced in the cytosol compared with nucleus **(C)** Within cytoplasm the number of clusters placed in 100 nm sided squares within or close to mitochondria up to a 250-nm distance from mitochondrial outer membrane, markedly increases after lithium exposure (**D** and Figure 1). In fact, in cells treated with lithium within 250 nm there is three-fold amount of grain clusters compared with vehicle treated cells. The ratio between mitochondrial grain clusters within 250 nm around mitochondria and cytoplasmic grain clusters significantly increases after lithium exposure **(E)**. Values are the mean ± S.E.M. Comparisons between groups were made by using One-way ANOVA (**C**) and Student's *t*-test for unpaired data **(A, B, D and E)**. ^*^P ≤ 0.05 compared with control. Scale bars: (low magnification) = 1.1 μm; (insert) = 0.43 μm.

Based on cell imaging with mitotrack red and green and the quantitative analysis of mitochondrial genes by rtPCR we already indicated that lithium induces the biogenesis of mitochondria (Fornai et al., 2008a). In line with this, a few weeks ago evidence was provided that autophagy induction is dually bound to mitophagy and mitochondriogenesis (Palikaras et al., 2015a,b). However, no single *in situ* morphological evidence was ever produced to document the occurrence of mitochondriogenesis under the effects of lithium. Thus, in the present study we pre-administered lithium chloride (1 mEq/L) or vehicle which was followed at 48 h by administration of [2-^3^H]adenine (1 μCi). In these experimental conditions, which follow up experimental data reported in Figure 1 and already commented in the Materials and Methods section we studied the distribution of autoradiography grains. As reported in representative Figure 13, these grains appeared as electrondense clusters of particles which were distributed in the cells very differently depending on exposure to vehicle or lithium. In detail, as shown in pictures of Figure 13 in a vehicle-administered cell the clusters distribute quite homogeneously in the cytosol, while a marked hetereogenity, polarized toward mitochondria was observed in the cytoplasm following lithium. As reported in Figure 1 this generates a hot area of [2-^3^H]adenine clusters which covers all the mitochondrial surface and the surrounding cytoplasm up to a 250 nm length from mitochondria. In this hot area, measurement of cluster density in homogeneous areas of squares measuring a side of 100 nm were similar. At this point, clusters density falls according to a geometrical distribution which confirms the model drawn by Salpeter (Salpeter et al., 1978). This represents the first *in situ* evidence for lithium induced mitochondriogenesis. The effects of lithium were evaluated on various patterns of radioactive grains. Lithium increases the number of grain clusters (Figure 13A); lithium increases the mean diameter of clusters (Figure 13B); lithium increases the polarization of cytoplamic vs. nuclear clusters (Figure 13C); lithium increases the number of clusters within and immediately around (250 nm) mitochondria (Figure 13D); lithium increases the ratio between mitochondrial and scattered cytosolic clusters (Figure 13E). This is in line with the curve we measured in Figure 1 for [2-^3^H]adenine distribution in the cytosol showing that, the distance of 250 nm corresponds to the critical length for which the highest difference is observed between lithium and vehicle-treated cells. At this distance, in lithium treated cells the number of grain clusters is still the highest and corresponds to the number of grain clusters counted on the mitochondrial surface. This is clearly visible in Figure 1 where the *Y*-values for radioactive clusters counted at *X* = 0 (corresponding to the mitochondrial surface) was constant in a range of *X* = 0–250 nm. This curve of cluster distribution produced by lithium was abolished in vehicle-treated cells This indicates significantly the occurrence of mitochondriogenesis according to the model of Salpeter et al. (1978). As described in the method the counts ruled out the staining produced by nuclear DNA.

**Figure 14:**
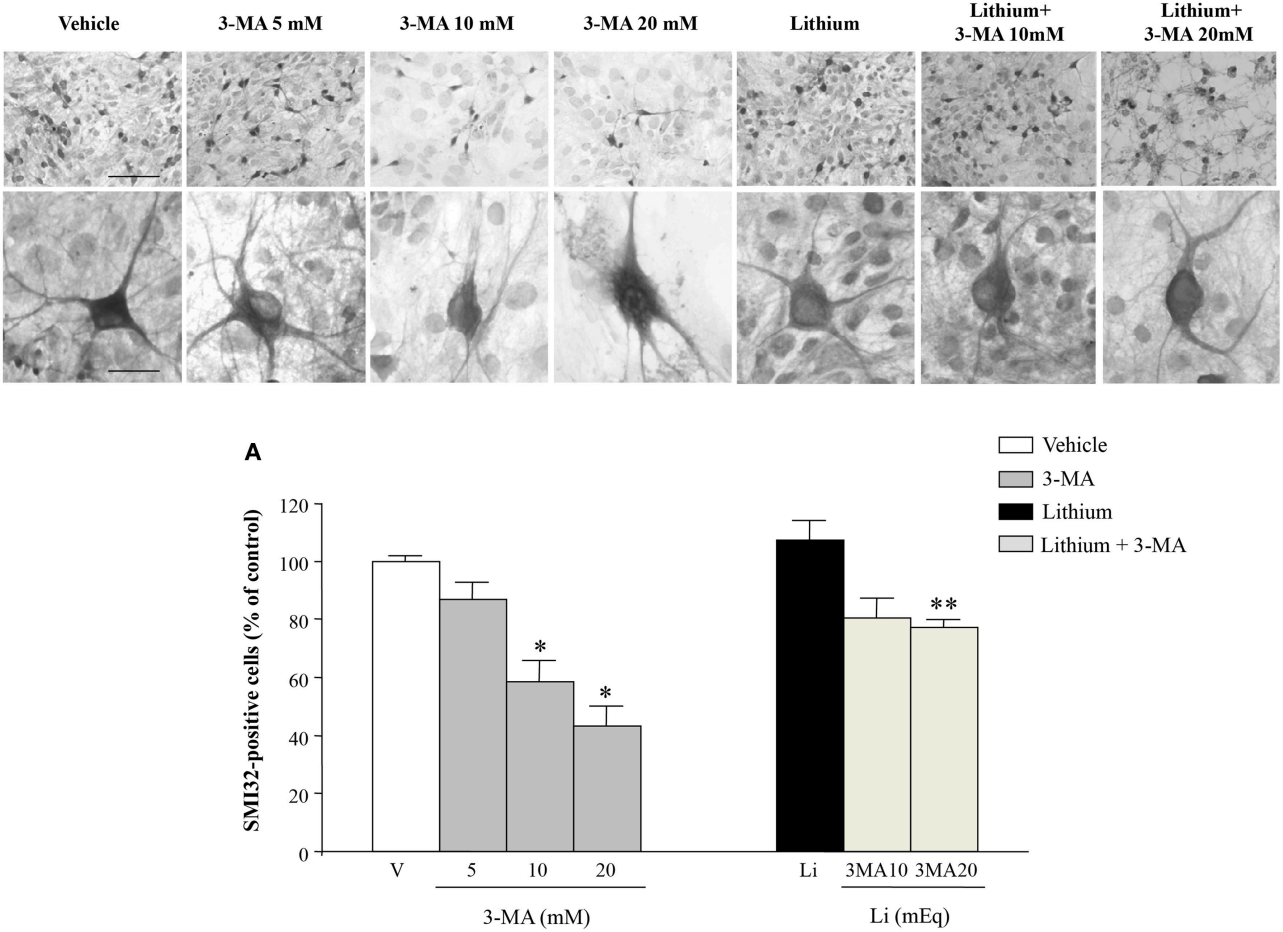
**Representative pictures at light microscopy primary motor neuron cultures from spinal cord after autophagy inhibition and stimulation**. Immunopositive SMI32 motor neurons were treated with the autophagy inhibitor 3-methyladenine (3-MA) at the doses of 5, 10, and 20 mM. As shown in representative pictures, 3-MA induces a dose-dependent decrease in the number of motor neurons. 3-MA also produces changes in the morphology of surviving motor neurons. Thus, witnessing for an autophagy-dependency of motor neuron survival. This effect is counteracted by the concomitant stimulation of the autophagy pathway by lithium at the dose of 1 mEq/L. These data were reported in graphs plotting the 3-MA-induced reduction in motor neuron number (**A**, left) and the protective effects of lithium (**A**, right). Values are the mean ± S.E.M. Comparisons between groups were made by using One-way ANOVA. ^*^P ≤ 0.05 compared with other groups in graph; ^**^P < 0.05 compared with 3-MA 10, 20 mM, and lithium **(A)**. Scale bars = 91 μm, high magnification: 19 μm.

**Figure 15:**
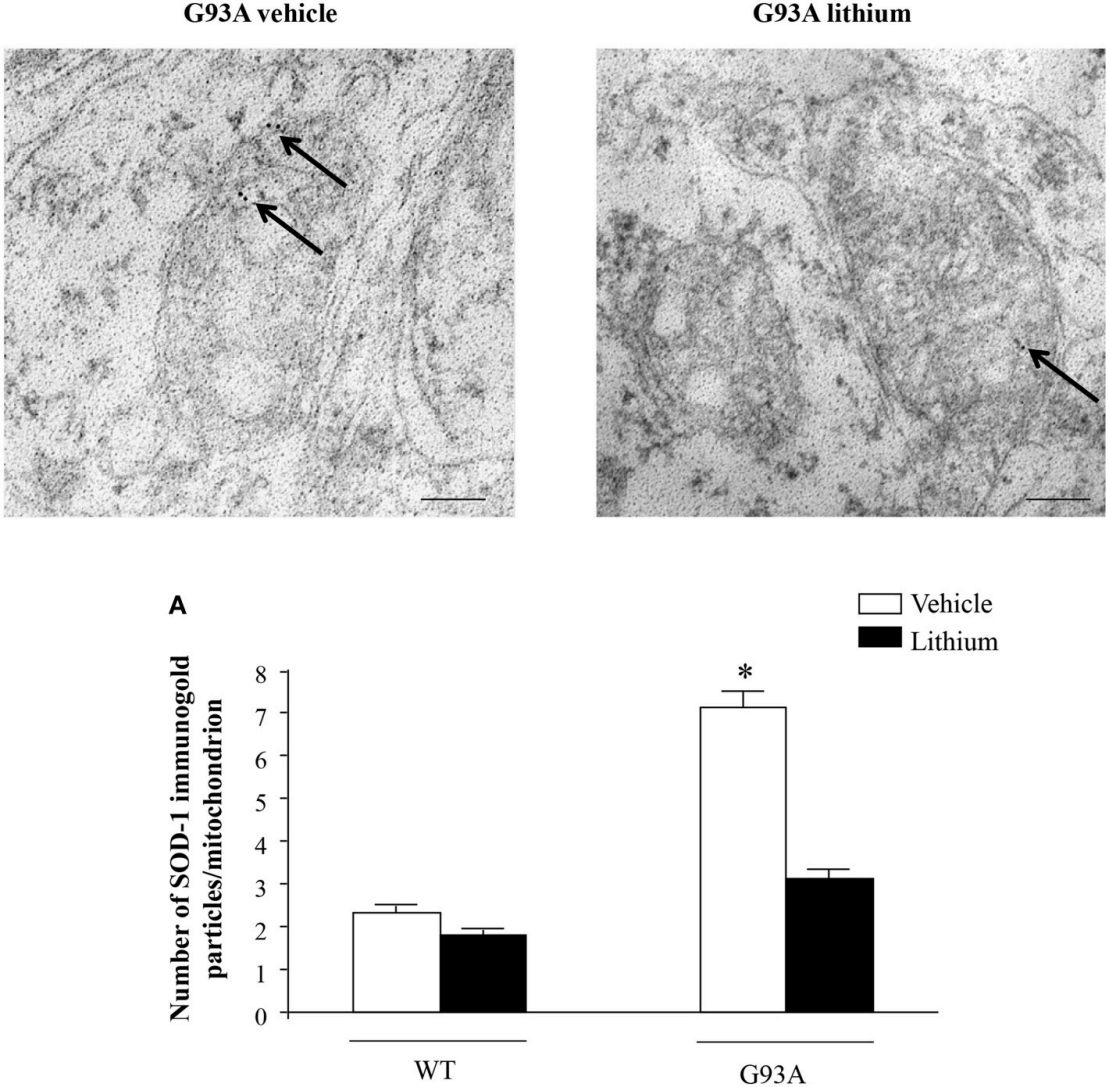
**Representative localization of SOD1 in motor neurons in G93A mice**. In vehicle-treated G93A mice SOD1 immunogold particles (arrows) are localized on the mitochondrial cristae and matrix. Lithium treatment significantly reduces the immunogold labeling (arrows, **A**). Values are the mean ± S.E.M. Comparisons between groups were made by using One-way ANOVA. ^*^P ≤ 0.05 compared with other groups. Scale bars: WT vehicle, WT lithium = 0.3 μm, G93A vehicle, G93A lithium = 0.4 μm, G93A vehicle (low magnification) = 1.3 μm.

In order to further confirm the role of autophagy in motor neuron survival and the autophagy-dependency of most part of lithium-induced neuroprotection we analyzed motor neuron survival in a very controlled experiment where primary motor neurons from the spinal cord were selectively exposed to an autophagy inhibitor. As shown in Figure 14, we administered the autophagy inhibitor 3-MA. When the highest dose of 3-MA was administered, SMI32 positive motor neurons were lost dramatically. Thus, in this *in vitro* model of ALS we could document autophagy-dependent motor neuron loss which was rescued by lithium administration (Figure 14). In these experimental conditions the subcellular changes replicated those described *in vivo* as well as the ultrastructural effects produced by lithium. Interestingly, as shown in representative pictures and graph of Figure 15, when we evaluated the immune-electron-microscopy of the protein SOD-1 we documented the occurrence of a lithium-dependent clearance of the SOD-1 protein. This effect confirms the ability of lithium to exert a direct protective effect at mitochondrial level by removing toxic mutated G93A SOD-1. Remarkably, while lithium-induced removal of G93A SOD-1 was significant, no noticeable effect was documented for WT SOD-1 (Figure 15). Additional effects were produced by lithium on glial cells as reported in the Supplementary Material (Figure S1) where lithium which is known to inhibit glial cells activation still re-shapes mitochondrial size. This effect does not relate to mitochondrial swelling which we could not document in glia at significant level.

## Discussion

In the present study we compared different motor neuron compartments in G93A SOD-1 mice. In each compartment we analyzed the mitochondrial pathology as well as extra-mitochondrial alterations. These studies were carried out both in baseline conditions and after autophagy induction in ALS mice compared with WT. Autophagy was induced by lithium administration which promoted effective progression of the autophagy pathway as documented in a variety of experimental studies as well as in the present work and in the Autophagy Guidelines by Klionsky et al. (2012). The analysis of motor neuron compartments was detailed at the level of dendrites, cell bodies and axons. The axonal compartment was further divided in proximal vs. distal axons. In this way we wish to evaluate whether the status of motor axons upstream in the spinal cord really reflects the ultrastructure of peripheral axons distributed in the muscle which are known to be early and severely damaged in ALS. In fact, when we measured mitochondrial alterations as well as other ultrastructural targets of ALS-related pathology we documented remarkable differences in distal compared with proximal axons. These differences are compatible with the disruption of axonal transport of mitochondria which was recently described by Magrané et al. (2012, 2014). In keeping with this, we documented here for the first time, the presence of axonal jams which clog significantly the axonal lumen suggesting a morphological correlate for altered mitochondrial transport along axons (Magrané et al., 2012, 2014). These axonal jams were hardly deciphered in their structure but they appear to contain mitochondrial remnants as well as proteinaceous aggregates. Significantly, when G93A mice were administered lithium, this compound cleared the axonal lumen and produced a significant improvement of mitochondrial number and morphology which was much more evident in the distal compared with proximal segment of the axons. These data confirm the seminal role of an impairment of autophagy in the progression of ALS. The occurrence of defective autophagy in ALS was demonstrated for the first time in the last decade (Fornai et al., 2008a,b). This was progressively validated through a growing number of studies (Pasquali et al., 2009; Ferrucci et al., 2010; Otomo et al., 2012; Shimada et al., 2012; Wang et al., 2012; Castillo et al., 2013; Ikenaka et al., 2013; Barmada et al., 2014; Cheng et al., 2015; Lee et al., 2015; Philips and Rothstein, 2015; Xiao et al., 2015; Yang et al., 2015) up to the very recent confirmative work by Xie et al. (2015a,b). This evidence was also reviewed in this issue by Ruffoli et al. (2015). In order to evaluate directly the significance of impaired autophagy for motor neuron survival, in the present study we administered the autophagy blocker 3-MA, which produced a dose dependent motor neuron loss, which in turn, was rescued by lithium. In the present study we extended the structural and morphometrical analysis to the gastrocnemius muscle which possesses dramatic light and electron microscopy alterations in G93A mice which were reverted by lithium. While the effects of lithium on motor neuron cell body were already reported, the occurrence of a remarkable effect in the distal vs. the proximal axons as well as in muscles was reported here for the first time in ALS mice. Interestingly, lithium is known to protect against a variety of peripheral neurophathy as shown in recent and classic literature. In fact, lithium prevents peripheral neuropathy induced by paclitaxel (Mo et al., 2012; Pourmohammadi et al., 2012) as well as the neuropathy induced by vincristine (Alimoradi et al., 2012) and there is evidence showing that lithium may even reverse the neurological damage induced by vinca alkaloids (Petrini et al., 1999). Interestingly, these compounds are known to inhibit vesicle trafficking, thus impairing autophagy progression and axonal transport. Again, in keeping with the dramatic morphological effects of lithium on peripheral nerves described here, previous studies indicate that lithium improves peripheral nerve regeneration after injury (Nouri et al., 2009) and it promotes axonal re-growth after ventral root avulsion (Fu et al., 2014). Similarly to peripheral nerves, muscles are known to be significantly affected by lithium which protects from a variety of degenerative changes such those occurring after muscle accumulation of amyloid beta and tau protein and muscle atrophy due to peripheral denervation (Askanas and Engel, 2008; Du et al., 2008; Kitazawa et al., 2008; Terracciano et al., 2010; Askanas et al., 2012; Fu et al., 2014; Su et al., 2014). Additionally lithium promotes myogenic differentiation and muscle regeneration (Polesskaya et al., 2003; van der Velden et al., 2007). The amount of muscle protection observed here along with a remarkable preservation of the sarcomeric architecture is similar to what demonstrated in these previous studies carried out administering lithium to muscles in pathological and baseline conditions. This study provides the first evidence describing a remarkable protection of muscle degeneration induced by lithium in the course of motor neuron disease. The protective effects induced by lithium were analyzed here by light end electron microscopy and they were measured by using morphometry of multiple targets within muscle cells. In fact, following lithium administration the dramatic loss of alignment of muscle fibers was prevented. Again, the abnormal inter-sarcomeric area was reduced back to WT values and the architecture of each sarcomer, which was dramatically altered, was largely reinstated. Similarly, the abnormal swelling of the sarcoplasmatic reticulum within muscle fibers from G93A mice was no longer present when mice were administered lithium. These effects, which are deeply grounded on the autophagy stimulating effects of lithium may also depend on a frankly primary protection by lithium of mitochondrial alterations such as altered calcium homeostasis. In fact lithium was shown to mitigate cytosolic calcium elevation (Bosche et al., 2013). Similarly, as shown by Scheibye-Knudsen et al. (2012), in the Cockayne syndrome type B mitochondrial protection is produced when gold standard autophagy activators such as lithium and rapamycin are administered. In the present study we did not assess directly potential neuroprotective effects exerted by lithium on mitochondrial activity; nonetheless we evaluated whether lithium was able to remove mutated SOD-1 from mitochondria, which in turn, is considered one potential mechanism of mitochondrial toxicity in ALS due to a G93A SOD-1 mutation. Other protective mechanisms exerted by lithium on mitochondria as a result of lithium-induced modulation of mitochondrial bioenergetics cannot be ruled out as convergent mechanisms to produce the overall beneficial results described here. Since the G93A mouse model is reported to suffer from an excess of mitochondrial accumulation of the mutant enzyme G93A SOD-1 within mitochondria, we evaluated the removal of this enzyme in lithium administered G93A mice. In fact, we documented *in situ* by representative pictures and morphometrical counts the removal of mutant but not WT SOD-1 in lithium administered G93A mice. This confirms at mitochondrial level the clearance of SOD-1 produced by lithium we already reported using western blotting (Fornai et al., 2008a). The present study provides significant evidence of a compartment-dependent damage to mitochondria and other sub-cellular structures in ALS, while demonstrating the compartment-specific neuroprotective effects of lithium. The removal of mutant SOD-1 from the mitochondria of lithium administered G93A mice is expected to lead indirectly to a preservation of mitochondrial architecture. This effect which was shown in the cell body of motor neurons (Figure 15), is still effective in the proximal axons, where it may contribute to explain why, in the absence of an effective mitochondrial turnover, the few mitochondria preserve their structure.

When analyzing at TEM the spinal cord of G93A mice, mitochondrial swelling and mitochondria-containing large stagnant vacuoles were measured within motor neuron perikarya and dendrites of lamina IX. These vacuoles were identified as authentic autophagy vacuoles by the gold standard (electron microscopy for multi-membrane vesicles staining both for LC3 and beclin I). Giant swollen mitochondria were characterized by severe dilution of the matrix and cristae breaking. The increase in mitochondrial size was dramatic and it was accompanied by an increase in the amount of altered mitochondria. This was associated with a dramatic filling with mitochondria-containing stagnant autophagy vacuoles within cell bodies of motor neurons. These effects confirm previous findings indicating a defective autophagy flux in the spinal cord both in ALS patients (Sasaki, 2011) and experimental ALS models (Fornai et al., 2008a; Li et al., 2008; Xie et al., 2015a,b). In these experimental conditions we analyzed the effects of a gold-standard autophagy inducer, which is lithium (Scheibye-Knudsen et al., 2012). Lithium is known for a long time as a mood stabilizer and neuroprotective agent (Madeo et al., 2009; Luo, 2010; Shimada et al., 2012; Chiu et al., 2013; Agam and Israelson, 2014; Lieu et al., 2014; Mohammadianinejad et al., 2014; Motoi et al., 2014; Yáñez et al., 2014; Dwivedi and Zhang, 2015) and it represents a classic autophagy inducer which was validated along several studies (Sarkar et al., 2005; Sarkar and Rubinsztein, 2006; Fornai et al., 2008a; Pasquali et al., 2010; Motoi et al., 2014) including the guidelines on autophagy (Klionsky et al., 2012), which represent the reference point for studies on the autophagy pathway. We found that lithium administration reverses/prevents almost completely alterations of mitochondrial architecture, the occurrence of swollen mitochondria and the presence of big stagnant autophagy vacuoles. The effects of lithium on mitochondrial size, shape, number and architecture are already evident in representative semi-thin sections and they were quantified by ultrastructural morphometry. Interestingly, the mitochondrial re-shaping effects produced by lithium were not limited to diseased motor neurons of G93A mice since we could document a significant mitochondrial plasticity induced by lithium even in motor neurons from WT mice.

Interestingly, while in vehicle-administered G93A mice we never documented the occurrence of small electron-dense mitochondria, within motor neurons of G93A mice administered lithium the size of mitochondria was markedly reduced and these organelles possessed an electron-dense ultrastructure, which generally corresponds to newly synthesized organelles. In G93A mice treated with lithium giant mitochondria were suppressed and the fine mitochondrial ultrastructure was preserved with slighter matrix dilution and rare cristae fragmentation being similar to controls. In fact, when counting mitochondria in both G93A and WT mice treated with lithium we documented the reduction in mitochondrial diameter, the increase in mitochondrial electron-density, and the increase in the total number of mitochondria. These small electron-dense mitochondria were barely detectable within autophagy vacuoles. Thus, following lithium mitochondria within cell bodies of motor neurons increased their number, but they also preserved a fine architecture and appear as electron dense well-structured small organelles. These effects are reminiscent of what happens following mitochondriogenesis. In fact, the stimulation of the biogenesis of mitochondria as well as mitochondrial fission increases the number of these organelles. However, when mitochondriogenesis is increased, the new mitochondria are very small electron-dense and well-conformed organelles, whereas the sole fission produces an increased number of mitochondria which otherwise appear of regular size and conformation. These data lend substance to what previously documented (Fornai et al., 2008a,b) suggesting that lithium produces mitochondriogenesis. In previous studies we provided such an evidence by measuring quantitative rtPCR for mitochondrial genes or MitoTracker Red and Green (Fornai et al., 2008a). This is in line with very recent data published by Palikaras et al. (2015a,b) showing the molecular mechanisms which eventually lead to a concomitant activation of mitophagy and mitochondriogenesis. In detail, when mitophagy is induced by autophagy activators, a molecular cascade is triggered which leads to a dual effect inducing concomitantly the biogenesis of novel mitochondria (Palikaras et al., 2015a,b).

In order to detail the occurrence of mitochondriogenesis *in situ* and to investigate these effects in other cell types, we measured the effects of lithium chloride in PC12 cells with or without pre-administration of the nucleotide precursor [2-^3^H]adenine *in vitro*. In this way, despite confirming a strong mitochondrial plasticity induced by lithium in baseline conditions, we could document by *in situ* morphometry that lithium produces a strong biogenesis of mitochondria as measured by incorporation of [2-^3^H]adenine. In detail, the administration of lithium to otherwise normal PC12 cells significantly modifies mitochondria, which is reminiscent of what observed *ex vivo* within motor neurons of WT mice treated with lithium. In fact, lithium administration further improved the architecture of mitochondria in PC12 cells. In particular, in vehicle administered PC12 cells, regularly shaped mitochondria with a slight degree of matrix dilution were observed. When PC12 cells were exposed to lithium mitochondrial morphology was well-defined with increased electron-density of the matrix and well-defined and densely packed cristae. Moreover, as described for *ex vivo* motor neurons' cell bodies, the number of mitochondria was dramatically increased following lithium exposure. This effect also extended to mitochondrial size, which was decreased by lithium administration. As previously mentioned, the occurrence at a high rate of small, well-structured, mitochondria suggests an ongoing mitochondriogenesis. Therefore, we measured directly *in situ*, by using TEM combined with [2-^3^H]adenine exposure, the occurrence of mitochondrial DNA replication. In detail, control and lithium-exposed PC12 cells were examined by high resolution autoradiography, following administration of [2-^3^H]adenine. Under these experimental conditions, radioactive labeling appears associated both with nucleus and cytoplasm. However, following lithium administration a significant increase in the number of grains was detected in the cytoplasm. [2-^3^H]Adenine was clustered specifically in very small areas surrounding mitochondria (which is compatible with grains diffusion constant established here: within mitochondria up to 250 nm distance from mitochondrial contour). In keeping with this, when lithium was administered, the distance between [2-^3^H]adenine labeling silver grain clusters and mitochondria was polarized within a range of 250 nm compared with stochastic diffusion which was measured in vehicle-treated cells (see Figure 1 again). Moreover, the mean size of electron microscopy clusters surrounding mitochondria was way higher in lithium compared with vehicle-treated cells. These data provide for the first time an *in situ* mechanistic evidence for mitochondrial biogenesis, thus lending substance to previous results we obtained by using quantitative rtPCR or MitoTracker Red or Green (Fornai et al., 2008a). This confirms that autophagy inducers which work as mitophagy activators produce a concomitant stimulation of mitochondriogenesis (Palikaras et al., 2015a,b).

In these latter works the mitophagy flux was coupled with the biogenesis of mitochondria. In fact, although autophagy induction was already suggested to be tightened with mitochondriogenesis (Fornai et al., 2008a; Ruffoli et al., 2015), very recently, elegant data indicated how mitophagy is coupled with biogenesis of novel mitochondria (Palikaras et al., 2015a,b). When damaged mitochondria occur even in physiological conditions, this triggers a dual pathway based on SKN-1 activation, which mediates mitophagy (the removal of damaged mitochondria), and increases mitochondrial biogenesis (Palikaras et al., 2015a,b). Interestingly, these dual effects were recently demonstrated for another autophagy inducer such as resveratrol (Meira-Martins et al., 2015), which is known to protect in ALS models (Han et al., 2012).

The significance of the present findings on motor neuron perikarya needs to take into account what recently published by Parone et al. (2013), who found that protecting mitochondria within motor neuron cell body and axons in the ventral root (through knocking out CycD thereby inducing the stabilization of the mitochondrial transition pore), does not extend the survival of G93A mice, despite preserving the number of motor neurons counted within spinal cord. The occurrence of palsy and lethality in these experimental conditions is considered to be the consequence of the axonal degeneration which still persists producing muscle denervation despite mitochondrial protection (Parone et al., 2013).

If one assumes that even axonal damage is produced by mitochondrial dysfunction, then it is likely that the threshold for mitochondrial damage in the cell body is higher than axons. This would explain why treatments leading to a noticeable protection of mitochondria in the motor neuron cell body do not guarantee for the survival of the peripheral axons. This is in line with mice undergoing palsy and death despite a fair sparing of motor neurons counted in the anterior horn of the spinal cord (Parone et al., 2013). Within this scenario we extended our analysis considering the effects of lithium within motor neuron axons. Here we noticed a substantial difference of mitochondrial damage in the proximal compared with distal axons and with what observed in the cell body. In the study by Parone et al. (2013), the occurrence of lethality and neurological symptoms was not modified by preserving mitochondria in the cell body and proximal axons ruling out the hegemonic role for mitochondrial alterations in the genesis of ALS. However, in this study axonal mitochondria were analyzed only at the level of the spinal cord/ventral root which means at the level of proximal axons. In the present study a clear difference was observed in mitochondrial degeneration and protection when considering proximal compared with distal axons. In particular, the amount of damage at mitochondrial level and within extra-mitochondrial structures in distal axons exceeds the damage which can be measured in proximal axons and cell bodies and dendrites. This indicates as mandatory to analyze the ultrastructure of peripheral axons in order to judge about the occurrence of axonal loss and muscle denervation, while proximal axons do not correlate with disease severity. In keeping with this, recent studies documented remarkable beneficial effects of lithium to enhance motor regeneration at axonal level (Fu et al., 2014; Su et al., 2014). This is in line with data reported here showing that lithium produces beneficial effects in peripheral axons and muscles. These effects are much slighter in proximal axons. The diversity of these axonal compartments may relate to the occurrence of axonal jams which clogs the axonal lumen which is cleared by lithium administration. The occurrence of axonal clogging is likely to make the distal axonal compartment no longer reachable by the cell body, unless a clearance of the axonal lumen is produced. In fact, the clearance induced by lithium of axonal lumen matches the increase in mitochondrial density and healthy mitochondria which was produced by lithium in distal axons. In keeping with the diversity of effects within proximal vs. distal axons some issues appear as contradictory. It may be difficult to explain why the amount of mitochondria counted in the proximal axon of lithium-treated WT is similar to lithium- and vehicle-administered G93A mice. In fact assuming a detrimental role of axonal jamming to impede mitochondrial axonal transport, one should expect that the number of mitochondria in lithium-treated WT was similar to vehicle-administered WT. Again G93A mice administered lithium should posses a number of mitochondria similar to that counted in G93A mice administered vehicle. However this point, despite being unexpected at first glance, confirms the profound difference between proximal and distal axon as distinct compartments in motor neuron pathology. In fact, when looking at distal axons, we measured the highest amount of mitochondrial damage and the presence of a significant protection by lithium. This is in line with the fact that proximal axons in the spinal cord do not represent the culprit of ALS degeneration which is instead localized within distant peripheral axons within muscles.

This is substantiated by the placement of axonal jamming which was detected upstream to distal axons and it was removed by lithium administration. Still, one might wonder why a lack of mitochondria in the proximal axon still occurs in lithium-treated mice. This is likely not to be a critical point for axonal physiology since, as in the cell bodies, proximal axons are not the site of neurotransmitter release and maximal energy consumption. These findings strengthen the introductory hypothesis of the manuscript that proximal compared with distal axonal damage is key to understand ALS pathology. Similarly functional data indicating impairment of axonal transport of mitochondria (Magrané et al., 2012, 2014) are now grounded on a solid morphological evidence provided here by axonal jamming. These data do not contradict the data obtained by Parone et al. (2013).

In fact in their study these Authors documented the preservation of mitochondria in the cell body and axons at the level of the ventral root (which means proximal axons), while the peripheral axonal degeneration and muscle denervation was still ongoing. In this study there was no ultrastructural analysis of mitochondria within peripheral axons where degeneration and denervation were taking place. The present findings shift the focus toward the very distal axonal compartment compared with proximal axons as key to understand the key role of mitochondria in producing axonal degeneration, muscle denervation and the course of ALS. It is likely that other neuronal alterations beyond mitochondria play a fundamental role in ALS; nonetheless the experiments reported here demonstrate that mitochondrial protection within distal axons is beneficial. Of course additional targets need to be considered. In fact, axonal clogging which occurs within axonal lumen is likely to play a critical role. Again, a frank beneficial effect on muscle ultrastructure shown here may improve the disease course also independently from motor innervation which remains fundamental. The protective effects of lithium described here on distal axonal mitochondria, axonal clogging and muscle derangement may not be powerful enough to arrest the disease course although they offer a solid proof of principle on how to target the critical points in the motor system which determine the onset and progression of motor symptoms as well as survival in ALS.

### Conflict of interest statement

The authors declare that the research was conducted in the absence of any commercial or financial relationships that could be construed as a potential conflict of interest.
