# Supplementary material for: Compartment-dependent mitochondrial alterations in experimental ALS, the effects of mitophagy and mitochondriogenesis
Source: Front Cell Neurosci. 2015 Nov 6;9:434. doi: 10.3389/fncel.2015.00434 (PMC4635226; doi:10.3389/fncel.2015.00434)
Supplement: Supplementary file 1 [file Image1.PDF]

Supplementary data

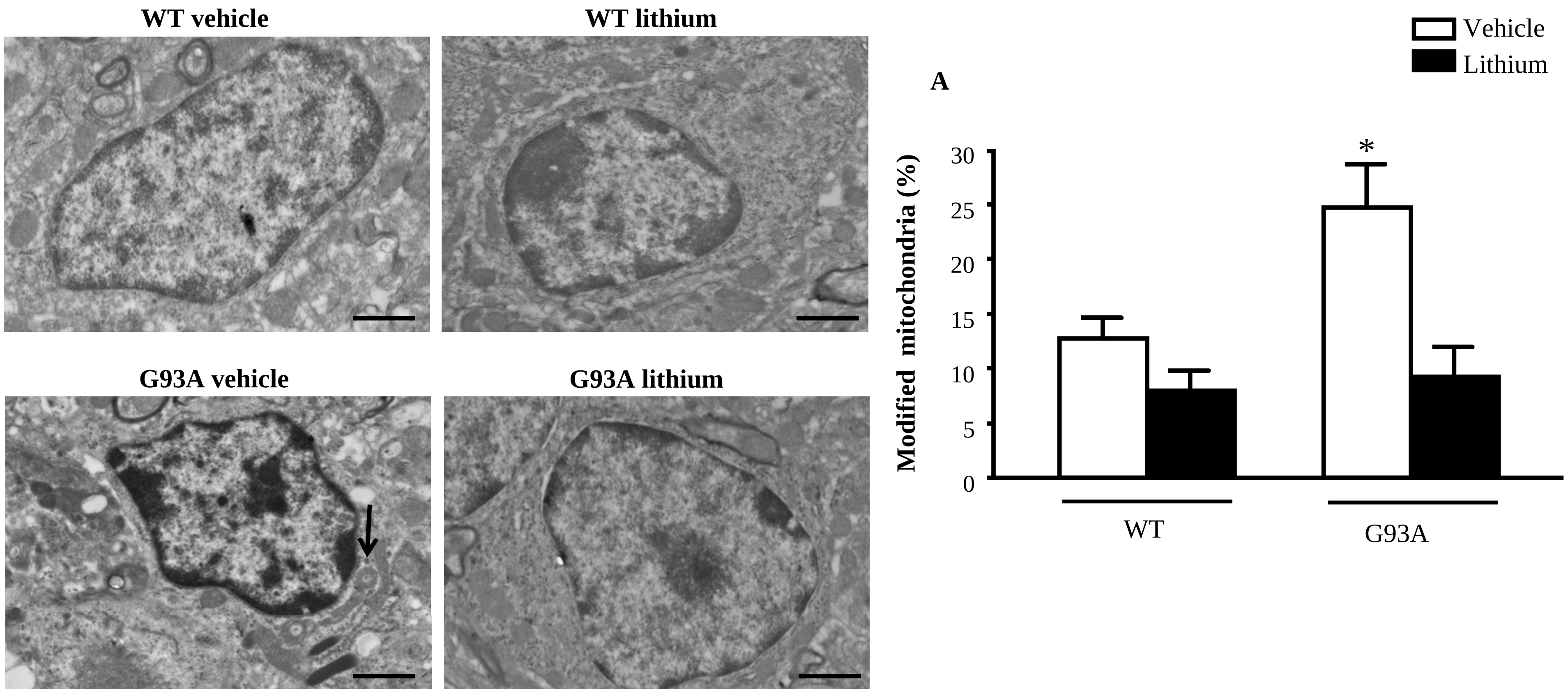

Figure S1. Representative pictures and morphometry of glial cells in anterior horn of spinal cord.
Electron micrographs of glial cells show a large nucleus with condensed patches of heterochromatin and a narrow cytoplasm containing scattered mitochondria. In vehicle-treated G93A mice modified mitochondria (arrow) are significantly increased with respect to other groups, as shown in graph **A**.
Values are the mean±S.E.M. Comparisons between groups were made by using one-way ANOVA. *P≤0.05 compared with other groups in graph (**A**).
Scale bars: WT lithium= 1 μm, WT vehicle, G93A vehicle and G93A lithium= 0.8 μm.

Figure S1
